# Deepfake-induced harm and AI accountability: a layered civil-liability framework for generative models, platforms, and digital identity

**DOI:** 10.3389/frai.2026.1873975

**Published:** 2026-07-02

**Authors:** Emad Ahmad Abousud

**Affiliations:** Department of Business Administration, College of Business Administration, Imam Mohammad Ibn Saud Islamic University (IMSIU), Riyadh, Saudi Arabia

**Keywords:** AI accountability, algorithmic causation, civil liability, Deepfake, digital identity, generative AI, Jordanian civil code, moral harm

## Abstract

Deepfake and other synthetic-media harms create a civil-liability problem that ordinary tort doctrine does not easily resolve: harmful content may be generated, amplified, monetised, and redistributed through a chain of actors in which no single participant controls the whole causal process. The objective of this article is to develop a layered civil-liability framework for that problem. It examines the Saudi and Jordanian civil-liability regimes as the principal doctrinal focus, while using selected EU, US, UAE, and Chinese materials as comparative reference points. The article remains private-law centred. It asks how civil-liability doctrine can respond when reputational, identity-based, corporate, and non-material harm is produced through the combined conduct of generative-model developers, prompting users, online platforms, and secondary distributors. The analysis identifies three pressure points: the natural-person wording of Article 138(2) of the Saudi Civil Transactions Law, which narrows moral-harm protection in relation to corporate and institutional injury; the persistence of a single-wrongdoer model in Saudi and Jordanian doctrine, despite the layered actor structure of deepfake production; and the evidential asymmetry created by opaque algorithmic causation. The article’s main contribution is a calibrated model of layered civil liability: clearer protection for juridical-person reputation, a custody-based rule for algorithmic systems, cautious burden shifting where relevant information is controlled by platforms or developers, stronger private-law links to data-protection regimes, and targeted transparency duties for synthetic-media systems. The contribution is not to replace civil law with AI regulation, but to show how both fields must work together if deepfake-induced harm is to be remedied in a legally coherent way.

## 1 Introduction

Deepfakes have moved beyond the stage of manipulated entertainment and isolated online abuse. They now form part of a wider problem of synthetic-media harm, capable of damaging personal dignity, corporate reputation, market trust, and public confidence in digital evidence. The legal difficulty is not simply that the technology is new. It is that the injury often emerges from a dispersed chain of design choices, user conduct, platform amplification, and further redistribution.

For conceptual clarity, this article uses the term “deepfake” in a legal rather than merely colloquial sense. Article 3(60) of the EU AI Act [Regulation (EU) 2024/1689, 2024] defines a deep fake as AI-generated or manipulated image, audio, or video content that resembles existing persons, objects, places, entities, or events and would falsely appear to a person to be authentic or truthful. This definition is useful here because it captures both generated and manipulated content and because it links the concept to deception of authenticity, which is the legally relevant feature for civil-liability analysis. The article adopts that definition as a comparative reference point, while acknowledging that Saudi and Jordanian civil-liability frameworks do not yet contain an equivalent statutory definition.

The January 2024 Hong Kong corporate-fraud incident, involving deepfake impersonation of senior executives (Hong Kong Police Force, 2024), captures the practical shape of the problem. It shows why deepfake harm should not be framed only around individual privacy or defamation. In commercially significant cases, the immediate loss may be financial, but the deeper injury may lie in institutional trust, reputational capital, and the reliability of digitally mediated identity.

This article addresses that problem through the Saudi and Jordanian civil-liability frameworks. The comparison is useful for two reasons. Saudi law has recently codified civil transactions in a comprehensive statutory form, while Jordanian law has long operated within the post-Sanhūrian Arab civilian tradition. Both systems share a civilian-Islamic structure, yet they diverge in ways that matter for deepfake liability: Saudi Article 138(2) expressly connects moral harm to the natural person, whereas both Saudi Article 132 (read with Article 134) and Jordanian Article 291 provide custody-based routes that may be adapted, cautiously, to algorithmic systems.

The legal dimension is therefore not ancillary to the article; it is its centre of gravity. AI governance is treated here as a necessary supplement to civil liability, not as a substitute for it (Henderson et al., 2023). A platform transparency rule, a data-protection remedy, or a synthetic-media label may reduce risk, but none of them answers the private-law question of who should compensate a victim once the harm has occurred.

The article therefore has a specific objective: to clarify how civil-liability doctrine should allocate responsibility when deepfake harm is produced through layered technical and human participation rather than through a single wrongdoer. Its contribution is both doctrinal and practical. Doctrinally, it identifies how Saudi and Jordanian private law can and cannot absorb deepfake-induced harm through existing categories of moral harm, direct and indirect causation, and custody of things. Practically, it proposes a layered liability framework that connects civil-law compensation with AI transparency, platform governance, data-protection remedies, and evidence-sensitive burden shifting.

The discussion proceeds in 12 sections. Section 2 states the research design and contribution. Section 3 maps the relevant literature and the remaining research gap. Section 4 explains the Saudi-Jordanian doctrinal architecture. Sections 5 to 7 analyse three gaps: claimant protection, layered actors, and algorithmic causation. Section 8 considers selected comparative frameworks. Section 9 explains the Sharī’ah foundation for protecting honour and reputation. Section 10 proposes reforms. Section 11 discusses why the problem persists despite existing legal tools. Section 12 concludes and identifies future research.

## 2 Research design, questions, and contribution

### 2.1 Research question

The principal question is how civil-liability doctrine should allocate responsibility for deepfake-induced harm when generative AI systems, human users, online platforms, and secondary distributors all contribute to the final injury, but none necessarily controls the whole process.

Three subsidiary questions follow. First, which parts of Saudi and Jordanian civil-liability doctrine can already respond to the practical profile of deepfake harm, and which parts leave victims under-protected? Secondly, what can be borrowed from EU, US, UAE, and Chinese responses without distorting the Saudi-Jordanian legal structure? Thirdly, what reforms would preserve the civil-law and Sharī’ah-sensitive character of the two systems while making liability more effective in algorithmic environments?

### 2.2 Working hypotheses

Four working hypotheses guide the article. They are not statistical hypotheses. They are doctrinal propositions tested through close reading, functional comparison, and assessment of how each legal rule would operate in a realistic deepfake dispute.

*H1 (Claimant-Protection Hypothesis). Saudi Article 138(2), by tying moral harm to the natural person, leaves a significant gap for corporate and institutional victims of deepfake reputational injury. Jordanian law is textually broader, but its judicial emphasis on psychological suffering may lead to a similar practical result.*

*H2 (Layered-Actor Hypothesis). Deepfake harm is poorly explained by a single-wrongdoer model. The Saudi direct-act causation rule and the Jordanian mubāshir-mutasabbib framework require supplementary reasoning when harm is produced through model developers, users, platforms, and further distributors.*

*H3 (AI-Governance Calibration Hypothesis). The EU combination of AI transparency duties, platform-risk obligations, and data-protection remedies offers more transferable guidance for Saudi and Jordanian law than a purely sectoral or piecemeal model, but only if adapted to local private-law concepts.*

*H4 (Normative-Compatibility Hypothesis). The Sharī’ah values of protecting honour, reputation, and dignity strengthen the normative case for broader protection against synthetic-media harm. In this article, however, those values operate as interpretive and legislative guidance, not as an independent substitute for statutory civil liability.*

### 2.3 Methodology

The article adopts a doctrinal-comparative method appropriate for a Policy and Practice Review in an interdisciplinary AI journal. It treats the Saudi Civil Transactions Law and the Jordanian Civil Code as the primary doctrinal materials, and reads them against selected comparative frameworks that address functionally similar problems of synthetic-media harm, platform amplification, and evidential asymmetry.

The empirical material is used in a deliberately modest way. Reported incidents, regulatory documents, and industry risk reports [Sumsub, 2024; World Economic Forum, 2024; Communications, Space and Technology Commission (Saudi Arabia), 2024; Arab News, 2025; Flynn et al., 2025] help identify the practical shape of the problem - corporate impersonation, reputational injury, viral redistribution, and evidential opacity. They do not convert the article into quantitative empirical legal research, nor do they displace doctrinal analysis.

The comparison is functional but cautious (Reimann, 2012). It asks what problem a foreign rule is designed to solve, whether the same problem exists in Saudi and Jordanian law, and whether the legal mechanism can be translated without damaging the coherence of the receiving system. The article therefore rejects both isolation from comparative law and uncritical legal transplantation.

One limitation should be made explicit. Published Saudi and Jordanian case law does not yet appear to include a direct civil-liability decision on deepfakes. The argument is therefore anticipatory. It seeks to clarify the issues courts and legislators are likely to face before doctrine hardens around unsuitable analogies.

This methodological design reflects the current state of the art. Much of the existing deepfake literature has examined privacy, defamation, election integrity, criminalisation, and platform regulation. Those perspectives are necessary, but they do not fully answer the private-law question of compensation once harm has occurred. The article therefore positions civil liability as the missing remedial layer in the broader AI-accountability debate.

The analysis proceeds in four phases. First, it defines the accountability problem and the relevant claimant interests. Secondly, it reads the Saudi and Jordanian civil-liability frameworks against that problem. Thirdly, it uses selected comparative models to identify transferable mechanisms rather than ready-made transplants. Fourthly, it converts the diagnosis into a reform model that preserves the internal coherence of Saudi and Jordanian private law.

## 3 Literature review: deepfakes, AI accountability, and civil liability

The literature on deepfake civil liability falls into four strands. Each has matured at a different pace and from a different starting point.^1^

### 3.1 US-centred deepfake-tort scholarship

The Anglo-American literature on deepfake civil liability begins with Chesney and Citron’s (2019) article in the California Law Review, which remains an important starting point.^2^ Chesney and Citron identified four threat dimensions—privacy, democracy, national security, market integrity—and proposed the “virality index” framework for damages calibration. Citron’s subsequent work with Solove on “privacy harms” extended the analysis to the broader category of digital privacy injury and articulated the evidentiary-asymmetry argument central to the third-gap analysis at section 7 below.^3^

The most comprehensive recent treatment is Kadri and West, 2025 article in the Journal of Tort Law, which provides a state-by-state survey of US tort and statutory frameworks and distinguishes the federal-criminal response from the emerging state-civil response. Delfino (2024) work extends the analysis into criminal procedure, identifying the “deepfake defense” problem in which any video evidence may be challenged as potentially synthetic (Delfino, 2024). Industry and policy surveys complement these doctrinal contributions.^4^^,^^5^^,^^6^

### 3.2 European AI-and-data-protection scholarship

The European literature is dominated by analysis of the EU AI Act and the Digital Services Act. Edwards’ summary of the AI Act (Edwards, 2024) and Bradford’s Digital Empires provide important English-language treatments of the regulatory architecture. Wilman’s commentary on the DSA (Wilman, 2022), the algorithmic-amplification analysis by Kohl (2024), the EU foundations articulated in Lohsse et al. (2019), and the recent commentaries on GDPR Article 82 jurisprudence supplement the regulatory analysis with the data-protection dimension.^7^^,^^8^

The civilian-doctrinal commentary on AI liability is led by Bacache (2020) and Loiseau (2019) in France, alongside Viney (2003) and Mazeaud et al. (1998); the recent CJEU jurisprudence—culminating in the Russmedia judgment of December 2025—has only just begun to attract sustained academic commentary.^9^^,^^10^

### 3.3 Asian comparative scholarship

Peng and Lee’s recent work in the Journal of Tort Law provides an important English-language treatment of the Chinese and Singaporean deepfake-tort frameworks. The Asian comparative material has not yet been integrated systematically into the Anglo-American literature.

### 3.4 Sharī’a-grounded scholarship on digital harm

The Sharī’a-grounded literature on synthetic-media harm is sparse but emerging. Three recent contributions bear on the present argument: a 2024 article in the Journal of Ecohumanism on Islamic legal analysis of online gender-based violence (Khotimah, 2024); a 2025 advance article on Islamic ethics frameworks for AI deepfake abuse (Uriawan et al., 2025); and a 2025 article in the Metro Islamic Law Review (Bawono et al., 2025) on the relevance of ḥifẓ al-nafs and ḥifẓ al-’irḍ to contemporary issues.^11^

These works treat *ḥifẓ al-’irḍ* as ethical principle rather than as the foundation of a specific civil-liability proposal. The present article extends their approach by linking the Sharī’a foundation explicitly to statutory-reform proposals.^12^ The Arab civilian-doctrinal literature on deepfake liability is, at the time of writing, even more limited.^13^ No published English-language article, as far as the available English-language literature indicates, has examined the Saudi Civil Transactions Law of 2023 against deepfake-induced harm with the specific focus on Article 138(2) developed at section 5 below.^14^ The Jordanian-specific literature is similarly thin.^15^

### 3.5 Position of the present article

This article contributes by treating deepfake civil liability as an AI-accountability problem with a private-law core. Existing scholarship has examined deepfakes through privacy, defamation, platform regulation, and criminal-law lenses. Less attention has been paid to how Arab civilian-Islamic civil codes would handle the same injuries, especially where the claimant is a company, the wrongdoer is anonymous, and the platform controls much of the relevant evidence.

The contribution is twofold. Doctrinally, the article provides a focused English-language analysis of Saudi and Jordanian civil-liability rules in the deepfake context. Conceptually, it proposes a layered liability model that links classical civil-law categories to the governance realities of generative AI systems.

#### 3.5.1 Novelty and remaining research gap

The novelty of the present article lies in bringing these strands together around a private-law question that has received limited attention: who should bear civil responsibility when deepfake harm is produced through generative models, human prompting, platform amplification, and secondary redistribution? The article does not treat Saudi Arabia and Jordan as merely local examples. It uses them as doctrinal test sites for a wider AI-accountability problem: whether traditional civil-liability frameworks can remain remedial in environments where causation, evidence, and control are technically distributed (Table 1).

**Table 1:** Research gaps and article contribution.

Existing literature	Remaining gap	Article contribution
US deepfake-tort scholarship focuses on privacy, defamation, publicity rights, intimate imagery, and sectoral statutory responses.	Limited attention to how Arab civilian-Islamic civil codes would allocate compensation for deepfake-induced harm.	Provides a Saudi-Jordanian private-law analysis centred on moral harm, actor attribution, and algorithmic causation.
EU AI-governance literature examines the AI Act, DSA, GDPR, transparency duties, and platform-risk obligations.	The link between regulatory duties and private-law compensation remains underdeveloped.	Connects AI transparency and platform governance to civil-liability mechanisms such as burden shifting and custody-based responsibility.
Sharī’ah-grounded scholarship addresses dignity, honour, and digital harm mainly at the ethical or normative level.	Limited translation of these values into targeted statutory civil-liability reforms.	Uses ḥifẓ al-’irḍ as interpretive and legislative guidance while preserving statutory civil-liability analysis.
Comparative AI-liability scholarship often treats platforms, users, and developers separately.	Insufficient account of deepfake harm as a layered chain of model design, prompting, amplification, and redistribution.	Develops a layered civil-liability framework that distributes responsibility according to control, foreseeability, and evidential access.

## 4 The Saudi-Jordanian doctrinal architecture

The question of whether existing Saudi and Jordanian civil-liability provisions can absorb deepfake-induced harm cannot be answered without first mapping the doctrinal terrain. This section provides that map. It argues that the two codes share a common civilian-Islamic structure, but diverge at two points that become decisive in deepfake disputes: the Saudi natural-person limitation for moral harm and the Jordanian custody-of-things doctrine.

### 4.1 Two codes, shared inheritance

Before assessing how each framework responds to the synthetic-media problem, it is useful to recall their shared lineage. The Jordanian Civil Code (Jordan) (1976) drew its civil-wrong provisions principally from the Egyptian Civil Code (Egypt) (1948)—itself the product of ‘Abd al-Razzāq al-Sanhūrī’s synthesis of French civilian doctrine with classical Islamic jurisprudence (al-Sanhūrī, 1952; Suwwār, 1996; Zakī, 1990; Mūsā, 2020), primarily Ḥanafī and Mālikī. The Saudi Civil Transactions Law, by contrast, is a 2023 enactment that codifies for the first time a body of obligations previously administered through Ḥanbalī jurisprudence and judicial discretion. Despite this generational gap, both codes share three structural commitments: a fault-based default for civil wrong, a shared civilian-Islamic concern with causation, although Jordanian law expressly codifies the direct/indirect harm distinction in Articles 257-258 while Saudi Article 121 adopts a narrower rebuttable causation presumption for acts attributed to their perpetrator.^16^^,^^17^^,^^18^

This shared inheritance carries methodological consequences for the present inquiry. Arguments developed in one jurisdiction frequently transfer to the other with only modest adjustment, which is why I treat them in parallel rather than sequentially throughout this article. It also means that whatever blind spots exist in one code are likely to find at least partial counterparts in the other—a hypothesis the analysis below confirms (Al-Ḥaqbānī, 2024).

### 4.2 The Saudi framework: an architecture of civil wrong

The Saudi Civil Transactions Law (2023) treats civil wrong (al-fi’l al-ḍārr) in Articles 119 through 145 (al-Mu’ajjal, 1442AH). The general clause, Article 119, provides that “any person who causes harm to another without right shall be liable to indemnify that harm”—a formulation that on its face echoes Article 1240 of the French Civil Code [Code Civil (France), 1804] (formerly Article 1382), but rests on a Ḥanbalī-Islamic substrate of ḍamān (guarantee/liability) rather than the French faute. Article 120 articulates the requirements of causation, while Article 121 does not reproduce the full Jordanian mubashir-mutasabbib framework. Rather, it establishes a rebuttable presumption that harm is causally attributable where the harmful act is attributed to its perpetrator.^19^^,^^20^^,^^21^

The codification of *mubāshir-mutasabbib* is consequential. It maps the chain of causation onto a binary that privileges proximity over substantive causal contribution—a structure inherited from the foundational Ḥanafī treatment of liability cascades.^22^ As I show in section 6 below, this binary will prove inadequate to the layered architecture of deepfake harm, where the “direct causer” may be a generative model, the “indirect causer” a platform, and the human content uploader an intermediate node of indeterminate doctrinal status.

Articles 122 through 124 address specific liability scenarios: liability of the keeper of an animal (Article 122), responsibility for the act of a subordinate (Article 123), and responsibility for buildings in disrepair (Article 124).^23^ Notably absent from this enumeration is any general doctrine of liability for things under one’s custody—an omission to which I return in section 6.

The 2023 Code’s most significant innovation for present purposes is Article 138, which for the first time codifies moral harm in Saudi statutory law.^24^ Article 138(1) provides that “compensation for civil wrong shall include compensation for moral harm.” Article 138(2), however, restricts the protected interest to “the natural person” (*al-shakhṣ dhū al-ṣifa al-ṭabī’iyya*), enumerating the heads of moral harm as physical or psychological suffering arising from injury to the body, freedom, honour, reputation, or social standing.^25^ Article 138(3) restricts the heritability of moral-harm claims, and Article 138(4) commits quantum to judicial discretion calibrated to “the nature of the moral harm and the person of the injured party.”^26^

Two elements of this architecture deserve early flagging. First, the natural-person restriction in Article 138(2) is a definitional choice with no direct parallel in either the Egyptian or Jordanian provisions on which Saudi codifiers might otherwise have drawn—an asymmetry I treat in detail in section 5.^27^ Second, the Code’s general framework operates alongside, rather than within, the Personal Data Protection Law of 2021, which provides a separate enforcement track for privacy-related harm but does not itself create a private right of action for moral damages independent of Article 138.^28^

### 4.3 The Jordanian framework: a wider doctrinal surface

The Jordanian Civil Code (Jordan) (1976) addresses civil wrong in Articles 256 through 292 (al-Zu’bī, 2018). Article 256 articulates the general principle: “any harm caused to another obliges its perpetrator, even if lacking discernment, to indemnify the harm.” Articles 257 through 266 elaborate the elements and qualifications of this liability, including provisions on concurrent fault and self-defence.^29^^,^^30^^,^^31^

Two provisions are important for the present inquiry. The first is Article 267, which provides that “the right to indemnity covers moral harm as well; thus any infringement on the freedom, honour, reputation, social standing, or financial standing of another renders the perpetrator liable to indemnify.”^32^ Unlike Saudi Article 138(2), Article 267 contains no natural-person restriction. On its face, it appears equally available to natural and juridical persons, with explicit reference to “financial standing” (*al-i’tibār al-mālī*) as a head of moral harm—wording particularly suggestive of corporate reputational interests.

The second important provision is Article 291, which establishes liability for things under custody:

Whoever has under his disposition things requiring special care to prevent their harm, or mechanical devices, shall be liable for the harm such things or devices cause, unless he proves that the occurrence of harm was due to a cause beyond his control.^33^

Article 291 derives, by way of the Egyptian Civil Code (Egypt) (1948) (Article 178), from the French jurisprudential development that culminated in the Jand’heur judgment of 13 February 1930 (Cass ch réun, 1930). Although its operative concept—la garde—is Romance in origin, its incorporation into the Jordanian code transposed it into a civilian framework that itself rests on Islamic foundations. The result is a doctrinal surface broader than that of the Saudi code: where Saudi law distributes liability primarily through the mubāshir-mutasabbib binary, Jordanian law adds a third doctrinal axis grounded in custodial relations to potentially harmful objects or systems.^34^

The Jordanian framework has also been augmented by recent legislation directly relevant to digital harm: the Cybercrimes Law (Jordan) (2023) and the Personal Data Protection Law (Jordan) (2023). Neither, however, provides a direct civil action for deepfake-induced harm, leaving the Civil Code as the principal repository of applicable doctrine.^35^

### 4.4 Convergence and divergence

In broad terms, the two codes converge on more than they diverge. Both treat fault as the default basis of liability. Both systems draw on the broader civilian-Islamic tradition of causation. Jordanian law expressly codifies the direct/indirect harm distinction in Articles 257-258, whereas Saudi Article 121 adopts a narrower rebuttable causation presumption for acts attributed to their perpetrator.^36^ Both recognise moral harm as compensable. Both leave damages quantum to judicial discretion subject to articulated criteria. And both operate against a regulatory backdrop of recent personal-data-protection statutes drawn closely on the GDPR template.^37^

The divergences, however, fall precisely where deepfake harm presses hardest. The natural-person restriction in Saudi Article 138(2) excludes from moral-harm protection an important category of victim—corporate entities—for whom synthetic-media reputational injury has emerged as the dominant litigation profile, exemplified by the Arup deepfake fraud of January 2024.^38^ No equivalent restriction appears in Jordanian Article 267. Equally, Saudi law contains a custody-based provision in Article 132, read with Article 134. The difficulty is not the absence of such a rule, but whether algorithmic systems and generative models can be treated as things requiring special care under that framework. Jordanian courts, in turn, retain the more explicit doctrinal possibility of treating a generative model as a “thing requiring special care”—a possibility I assess in section 6.

These two divergences, taken together, set the agenda for the rest of the article. The first underwrites the analysis of section 5; the second of sections 6 and 7. The comparative material in section 8 then asks which external models—EU, US, or the intermediate UAE framework—offer the most calibrated response to these gaps.

## 5 The first gap: moral harm and the natural-person limitation

### 5.1 The textual exclusion

The most important restriction in the Saudi Civil Transactions Law for deepfake liability does not announce itself in structural terms. It appears in a single phrase. Article 138(2) confines the moral-harm head of damages to “the natural person” (*al-shakhṣ dhū al-ṣifa al-ṭabī’iyya*), and that phrase, read against the established canons of Saudi statutory construction, excludes juridical persons from the protected category.^39^ The following analysis argues that this exclusion is doctrinally clear, difficult to bridge on the current text, and difficult to justify in the deepfake context.

The textual difficulty is substantial. Article 138(2) enumerates the protected heads—physical or psychological suffering, injuries to body, freedom, honour, reputation, and social standing—and anchors them all to the natural person.^40^ Three of those heads (suffering, body, freedom) presuppose a corporeal subject, providing the surface logic of the limitation. The remaining three (honour, reputation, social standing) apply with equal force to corporate entities. The provision draws the boundary at corporeality and then extends it outward to capture the non-corporeal heads as well. This produces a problematic definitional boundary: a category drawn coherently for one set of heads is conscripted to police others where its rationale does not extend.

The classical canon of “the specific qualifies the general” (*al-khāṣṣ yuqayyid al-’āmm*) substantially narrows the available interpretive routes.^41^ Article 119, the general clause on civil wrong, makes no distinction between natural and juridical claimants. Article 138, by contrast, is the specific provision governing moral harm. On Saudi interpretive principles, the specific reading prevails over any reading drawn from the general clause.^42^ Article 119 cannot be invoked to override the specific limitation in Article 138(2). Worth noting: the position the 2023 codifiers took here also represents a deliberate departure from the more open Ḥanbalī classical position, on which moral harm to corporate-style endowments and the reputational interests of charitable institutions was at least debatable.^43^

### 5.2 The Jordanian counterposition

Article 267 of the Jordanian Civil Code presents a textually broader provision. It speaks of “anyone whose freedom, honour, reputation, social standing, or financial standing” is infringed, with no parenthetical limitation to natural persons.^44^ The express reference to “financial standing” (*al-i’tibār al-mālī*) particularly suggests a legislative awareness that financial reputation—a head of injury most coherently asserted by a corporate entity—falls within the article’s protective scope.

Jordanian judicial practice, however, has not followed Jordanian text. The Court of Cassation has anchored the moral-harm provision to the subjective psychological experience of the victim—a construction that effectively excludes corporate plaintiffs from the head, since a corporate entity has no psyche to experience moral suffering.^45^ The Egyptian doctrinal tradition on which the Jordanian Code rests is itself ambivalent on this point, and that ambivalence has surfaced in Jordanian application of Article 267.^46^ The two regimes thus converge on outcomes from textually divergent starting points—a convergence that gives empirical force to Pound’s older observation that “law in action” can reverse “law in books.”^47^

This convergence carries analytical consequences. The corporate plaintiff in either jurisdiction faces the same effective bar: in Saudi Arabia, by clear textual exclusion; in Jordan, by entrenched judicial gloss. The two require different reform instruments—a textual amendment in Saudi Arabia, a doctrinal reorientation in Jordan—but they share an underlying gap that any responsible reform package must address simultaneously.^48^

### 5.3 The empirical profile of corporate deepfake harm

The natural-person limitation might be defensible if corporate deepfake harm were a marginal phenomenon. It is not. The one of the most significant emerging litigation profiles of synthetic-media injury is corporate, not individual. The Hong Kong Arup case of January 2024—in which a deepfake-enabled video conference impersonating senior executives induced fraudulent transfers (Hong Kong Police Force, 2024; Sumsub, 2024; Flynn et al., 2025) exceeding US$25 million—has become the touchstone for a category of harm that is recurrent, not exceptional. Sumsub (2024) Identity Fraud Report records a tenfold increase in deepfake-enabled corporate fraud over a single 12-month period. World Economic Forum threat assessments rank synthetic-media corporate impersonation among the leading emergent risks to the global financial system. Within the Gulf, the Saudi Communications, Space and Technology Commission and independent identity-verification analytics report a 600 per cent surge in deepfake incidents in the Kingdom in the first quarter of 2024 alone.^49^^,^^50^^,^^51^^,^^52^

These indicators matter for the doctrinal argument. They mean Article 138(2) does not merely affect a marginal category of plaintiff. It may affect a central category of claimant.

The corporate harm, when it materialises, is not merely an indirect derivative of injury to individual officers or shareholders. It is a sui generis injury to the corporate reputational interest—what economic and legal scholarship has begun to call “reputational capital.”^53^ A corporate entity targeted by sophisticated synthetic-media fraud suffers market-trust degradation, customer attrition, regulatory scrutiny, and elevated insurance premia, none of which is fully captured by the financial-loss head alone.^54^ Restricting the remedy to provable pecuniary damage forces the corporate plaintiff to litigate in a head of damages that systematically under-captures the harm. Recent CJEU jurisprudence on platform liability under the GDPR—most notably the Russmedia judgment of December 2025—has begun to acknowledge precisely this corporate-reputational dimension as a protected interest in EU law.^55^ The Saudi and Jordanian frameworks have not yet caught up.

### 5.4 Three interpretive escapes that fail

One might attempt three doctrinal routes around the limitation. Each has limits.

The first invokes Article 138(4), which grants the court discretion to calibrate damages “in light of the nature of the moral harm and the person of the injured party.”^56^ On a generous reading, “the person of the injured party” might be argued to admit corporate persons. But Article 138(4) governs quantum, not eligibility. It tells the court how to set damages once a recoverable head of harm is established. It cannot create a recoverable head where Article 138(2) excludes one.^57^

The second invokes Article 29, which prohibits abuse of right (*ta’assuf fī isti’māl al-ḥaqq*).^58^ The argument runs: a deepfake creator abuses a right by using a generative tool to harm another. But Article 29 presupposes the existence of an underlying right whose exercise crosses into abuse. The deepfake creator typically possesses no such underlying right—unauthorised impersonation is not the abuse of a right but the absence of one. Article 29 is therefore not the operative doctrinal frame.^59^

The third invokes the broad language of Article 119 (“any person who causes harm to another without right”), arguing that the general clause’s scope encompasses synthetic-media injury whether the claimant is natural or juridical. This route does not contradict Article 138(2) directly—it operates in a separate doctrinal channel—but it leaves the corporate plaintiff seeking compensation only for material damage, since the moral-harm head remains locked in Article 138.^60^ The corporate plaintiff thus recovers only what is demonstrably pecuniary, while the reputational-capital harm—for which the moral-harm doctrine exists—goes uncompensated. The contrast with the French position, where moral damages are available to legal persons under the general civil-wrong provision, underscores the deliberate character of the Saudi limitation.^61^

A fourth route might appear to be available through the Personal Data Protection Law (Saudi Arabia) (2021), since deepfake harm often involves processing of personal data. But the PDPL provides administrative and criminal mechanisms; it does not itself create a private cause of action for moral damages independent of the Civil Transactions Law’s general framework. The PDPL therefore cannot fill the Article 138(2) gap.^62^

### 5.5 The gap stated

The first gap is primarily structural, not merely interpretive. It cannot be cured by canonical extension of existing texts. It requires either textual amendment in Saudi Arabia or a doctrinal reversal of accumulated Jordanian case law.^63^ The two paths are unequal in difficulty: legislative amendment of a 2023 code, while requiring legislative will, is doctrinally cleaner than reversing two decades of high-court reasoning.^64^ Section 10 below proposes the textual route for both jurisdictions, with a calibration adapted to each.^65^

The next section considers the second gap. Even where moral harm is recoverable as a matter of doctrine, the chain of causation that produces deepfake injury cannot be mapped onto the singular tortfeasor that classical tort law presupposes. The architecture of synthetic-media harm has multiple authors. Classical tort law, in both its Saudi and Jordanian variants, has contemplated only one.

## 6 The second gap: the Tortfeasor as singular actor

### 6.1 The singular-Tortfeasor presupposition

When the Saudi and Jordanian codes articulate the foundational rule of civil wrong—Article 119 of the Saudi Civil Transactions Law and Article 256 of the Jordanian Civil Code—they speak in the singular. “Any person who causes harm to another”; “any harm caused to another obliges its perpetrator.”^66^ The textual frame contemplates one wrongdoer, one harm, one indemnity. This singular grammar is not accidental. It rests on a centuries-deep assumption—operative throughout classical Islamic jurisprudence and post-revolutionary French civilian doctrine alike—that a tortious act is performed by an agent whose conduct can be isolated, evaluated, and attributed.^67^

The assumption holds reasonably well for the paradigm cases that shaped the doctrine. A driver causes a collision. A neighbour’s wall collapses on adjoining property. A merchant deceives a customer. In each case, a single human agent’s conduct can be plausibly traced to the harm.^68^ But the deepfake is not such a case. The synthetic-media injury that this article addresses is produced through a sequence of acts performed by structurally distinct actors operating across temporal and institutional gaps that classical tort doctrine was not designed to traverse.

I argue in this section that the singular-tortfeasor presupposition is under strain in the deepfake context, and that Saudi and Jordanian doctrine respond through differently configured custody-based routes. Jordanian Article 291 expressly addresses things requiring special care and mechanical devices, while Saudi Article 132, read with Article 134, provides a custody rule based on effective control. The two systems therefore arrive at the same problem from different doctrinal starting points, not from the presence of a rule in one system and its total absence in the other.

### 6.2 The layered architecture of deepfake production

The production of a deepfake that reaches a victim involves at least four distinguishable layers of action.^69^

The first layer is the foundation model—the trained generative system whose architecture and weights make synthetic image, audio, or video generation possible. The developer of the foundation model has, in most contemporary deepfake events, no specific awareness of any particular harmful output.^70^ But the developer’s choices regarding training data, output filters, watermarking, and access controls shape the universe of possible misuse.^71^ The model is not a passive instrument in the sense that a hammer is passive. It encodes, through training, predispositions that downstream actors then exploit.

The second layer is the human prompter or uploader—the natural person who causes the model to generate a specific harmful output, or who edits and posts a generated artefact. This is the layer that most resembles the classical tortfeasor: a human agent making a particular choice with a particular victim in view. But this agent is often anonymous, often outside the practical reach of the victim’s jurisdiction, and frequently judgment-proof.^72^

The third layer is the platform that hosts and disseminates the artefact. The platform is not a passive bulletin board; its recommendation algorithms determine which content reaches which users and at what scale.^73^ Two cases of corporate impersonation may produce equivalent legal injury, yet the actual harm—measured in audience reach, persistence, and reputational damage—depends almost entirely on what the platform’s algorithm does with the content after upload.

The fourth layer is the secondary distributor—the user who screenshots, re-uploads, or repackages the content across platforms, often after the original is removed.^74^ This layer is what gives synthetic-media harm its characteristic durability: once a deepfake becomes viral, it acquires a half-life that no take-down order from any single platform can fully end.

These four layers are not always all present. A deepfake that does not propagate beyond a small audience may involve only the first three. Some categories of harm—particularly those targeting individuals known only locally—may turn principally on the second layer. But for the dominant litigation profile identified in section 5, all four layers are typically engaged, and the doctrinal question becomes how to distribute responsibility across them.

### 6.3 The limits of direct-act causation and direct/indirect attribution

The layered architecture exposes a causation-allocation difficulty. Jordanian Articles 257-258 articulate the direct/indirect harm distinction expressly, while Saudi Article 121 provides a narrower presumption of causation where the harmful act is attributed to its perpetrator. Neither formulation comfortably accommodates four structurally distinct actors.^75^

The candidates are not equivalent. If the human prompter is the *mubāshir*, the platform and the model developer become mere indirect causers—and Article 121’s default rule then assigns liability primarily to the (often anonymous, often judgment-proof) prompter, with the platform and developer escaping unless they “transgressed.”^76^ This is the prompter-as-mubāshir reading. It produces a doctrinally clean attribution, but at the cost of leaving the most culpable and most reachable parties—the platform and the model developer—outside the primary liability frame.

Alternatively, one might argue the model itself is the *mubāshir*, since it is the proximate cause of the synthetic artefact’s generation. But this reading conflates causation with agency, treating a non-agentic tool as a causally responsible party. Classical Islamic jurisprudence anticipated this problem in its discussion of the role of inanimate causes (*al-asbāb al-māddiyya*), and resolved it by attributing causation to the closest agentic actor.^77^ Under that resolution, the model is not the *mubāshir*; the prompter is. The binary thus collapses back into the first reading.

A third reading would attempt to dissolve the binary by treating all four layers as concurrent *mubāshirūn*. But this is not what Article 121 says, and there is no clear doctrinal authority within Saudi codified law for joint primary liability of structurally distinct actors in this manner.^78^

The result is that the *mubāshir-mutasabbib* framework, applied to the deepfake context, produces either: primary liability concentrated on the least reachable party (the prompter), with the platform and developer reduced to indirect-causer status; or a doctrinally strained attempt to read joint primary liability into a text designed for binary attribution. Neither outcome is doctrinally satisfactory.

### 6.4 Jordanian Article 291 and Saudi Article 132 as doctrinal bridges

Jordanian Article 291 and Saudi Article 132 both provide a possible doctrinal bridge for algorithmic custody. Jordanian law is explicit in referring to mechanical devices, while Saudi law is significant because Article 134 defines custody through actual authority over the thing. The article reads: “whoever has under his disposition things requiring special care to prevent their harm, or mechanical devices, shall be liable for the harm such things or devices cause.”^79^ On its face, this is a doctrine designed for physical objects—vehicles, machinery, animals.^80^ But its inner logic—that one who has *control* over a *risk-generating thing* bears strict liability for the harms that thing produces—extends naturally to algorithmic systems.

The doctrine has its modern Jordanian-Egyptian-French ancestry in the Jand’heur judgment of 1930 and the subsequent Franck judgment of 1941 (Cass ch réun, 1941), where the French Cour de cassation held that the liability of the gardien attaches not to physical possession but to the power of “use, direction, and control” (usage, direction, contrôle). This control-based criterion translates well to the foundation-model context: the developer who controls model architecture, training data, output filters, and access controls is the one whose decisions shape the field of possible harm. The platform that hosts and algorithmically amplifies content controls a different domain—the dissemination architecture—but its control is no less real.^81^^,^^82^

I do not suggest that either Jordanian Article 291 or Saudi Article 132 has yet been applied by courts to a generative-AI system or a platform content-distribution algorithm.^83^ But the doctrinal possibility is present, and recent French scholarship has begun to articulate the conceptual extension under the rubric of “*garde de l’algorithme*”—custody of the algorithm.^84^ What has so far prevented Jordanian courts from making the move is, in my assessment, less doctrinal obstacle than absence of opportunity. The cases have not yet arrived in number sufficient to force the issue.

### 6.5 The Saudi interpretive challenge

The contrast with the Saudi position is now stark. “Saudi law does contain a custody-based provision in Article 132, supported by Article 134. The difficulty is therefore not the absence of a Saudi custody rule, but its application to non-physical or hybrid algorithmic systems. Article 132 covers things requiring special care by nature or by legal provision; Article 134 defines custody by actual authority. These provisions provide a possible basis for algorithmic custody, but their application to generative AI requires careful judicial or legislative clarification.^85^ Article 119’s general clause, Article 121’s direct-act causation presumption, and, where custody is argued, Articles 132–134.

Two interpretive moves remain available to the Saudi court. The first reads Article 119’s “any person who causes harm” expansively to encompass the platform and developer as concurrent perpetrators of harm. This is a defensible reading, but it leaves quantum and apportionment unguided.^86^ The second analogizes to Article 122 (animal custody), treating the foundation model as a “thing requiring special care” by analogical extension.^87^ But Saudi doctrine on analogical extension within statutory interpretation is more cautious than its Jordanian counterpart.^88^ Both moves are doctrinally available. Neither is doctrinally clean.

The structural conclusion is the same as that of section 5: the gap is real, and interpretive routes around it are strained. Section 10 below argues that the appropriate response is the introduction, into Saudi law, of the clarification of Article 132 and Article 134 so that, where appropriate, high-risk algorithmic systems and generative-AI tools may fall within the custody-based framework.^89^ The comparative-models question—whether the EU’s risk-based platform duties under DSA Article 35, the US sectoral approach, or the Chinese personality-rights model offer better analogues for the Saudi-Jordanian context—is taken up in section 8.^90^ Recent US litigation has begun to recognise platform amplification as legally distinct from passive hosting,^91^ and the EU’s VLOP regime offers a structural template that aligns more closely with the doctrinal architecture of both Arab civil codes.^92^

## 7 The third gap: causation in algorithmic environments

### 7.1 The classical doctrines of causation

The Sanhūrian synthesis on which both the Saudi and Jordanian codes rest treats causation as a question of “the productive cause” (*al-sabab al-muntij*)—drawn from the French theory of *la cause adéquate*.^93^ On this view, the law selects from among the multiple antecedents of a harmful outcome the one (or the few) whose contribution was substantial enough to ground attribution. The selection is normative, not merely factual; it asks which cause the law treats as legally significant.^94^

This doctrine works best in a world of sequentially ordered events. A driver runs a red light; a pedestrian is struck; medical complications follow. Each link in the chain is identifiable; the direction of causal flow is intuitive; the foreseeability of the harm is assessable from the perspective of the actor at the relevant link. In such a world, the productive cause can be identified by trained judicial intuition.

The deepfake case is structured differently. The harm flows not from a sequential chain but from a system in which multiple causes operate concurrently and interact in ways that cannot be predicted from the perspective of any single actor.^95^ The model developer’s training-data choices, the prompter’s specific input, the platform’s recommendation algorithm, and the secondary-distribution dynamics all interact to produce the eventual harm. The interaction is not merely additive; it is multiplicative. Removing any one input materially changes the output.

### 7.2 The opacity of algorithmic causation

Classical causation doctrine assumes that, at least in principle, the chain of causation is reconstructable. The algorithmic environment defeats this assumption in three ways.

First, the operation of contemporary recommendation systems is not directly observable even by their operators.^96^ Engagement-optimised algorithms are tuned to maximise a target metric (time-on-platform, engagement rate) by adjusting weights in ways that produce emergent rather than designed outcomes.^97^ The platform engineer cannot, in advance of deployment, predict which deepfake the algorithm will surface to which users.

Second, the foundation model’s contribution to a specific output is similarly opaque. The model’s “understanding” is distributed across billions of parameters that do not correspond to interpretable rules.^98^ The developer can shape the distribution of likely outputs through training-data curation and output filtering, but cannot guarantee that any particular synthetic artefact will not be produced.

Third, the secondary-distribution layer—the human re-uploaders and screenshot-takers who carry harmful content beyond its original platform—operates outside any single chain of legal control. The harm propagates through a network in which the legally relevant question (who caused this propagation?) has no satisfactory single-actor answer.^99^

### 7.3 Three pressures on classical doctrine

These features impose three distinct pressures on the *al-sabab al-muntij* framework.

The first is the pressure of multi-causal contribution. Where the productive-cause doctrine asks “which cause was substantial?”, the deepfake case requires the answer “all of them.” But the legal consequence is unclear. Saudi Article 121’s direct-act causation presumption is unequipped to handle this answer; Jordanian Article 291 and Saudi Articles 132-134 may absorb it for the model and platform, subject to careful interpretation, but neither fully addresses the prompter or the secondary distributor.

The second is the pressure of foreseeability across structural distance. Foreseeability is calibrated, in classical doctrine, to the position of the actor at the moment of the act.^100^ The model developer, training the system years before any specific harmful use, cannot foresee the particular harm that a particular prompter will eventually engineer. Yet the developer is in a position to shape the universe of foreseeable misuses through technical safeguards.^101^ Foreseeability of the category but not the instance—and the legal question of whether category-level foreseeability triggers liability for instance-level harm—is a question the classical doctrine is poorly equipped to resolve.

The third is the pressure of evidentiary access. Even where the doctrine could in principle apportion liability among the layers, the evidentiary record needed to do so—model architecture, training data, recommendation-algorithm logs—is in the exclusive control of the defendants.^102^ The plaintiff cannot easily obtain it; production rules in Saudi and Jordanian civil procedure offer limited assistance against well-resourced corporate defendants.^103^

### 7.4 What the comparative frameworks suggest

The comparative material treated more fully in section 8 below offers two distinct responses to these pressures.

The EU response, articulated through Article 35 of the Digital Services Act and the recent CJEU jurisprudence in *Russmedia*, recasts causation as a regulatory rather than civil-procedure question.^104^ Very large online platforms are required to identify and mitigate “systemic risks”—including those associated with manipulated synthetic media—through risk-assessment procedures whose outputs are themselves regulated. Civil liability follows from regulatory failure rather than from classical causation analysis. The classical doctrine is not displaced; it is supplemented by a parallel regime that handles the multi-causal cases its causation analysis cannot.

The US response, by contrast, has been more fragmentary. Federal law currently provides no general civil cause of action for deepfake harm. The TAKE IT DOWN Act of 2025 creates federal criminal prohibitions and notice-and-removal duties for covered platforms in relation to non-consensual intimate visual depictions and digital forgeries, but it does not create a general private damages action for deepfake harm. The DEFIANCE Act of 2025, passed by the Senate in January 2026 and pending before the House at the time of writing, would provide a federal civil cause of action for victims of non-consensual intimate deepfakes. The general-purpose state-level civil-tort frameworks—defamation, false light, intentional infliction of emotional distress—apply only intermittently to deepfake cases, and Section 230 immunity continues to shield most platforms from causation-based liability outside the algorithmic-amplification context.

A counterfactual (“but-for”) test, which classical tort doctrine still operates as a baseline, is particularly ill-suited to the multi-causal deepfake setting.^105^ The test asks whether the harm would have occurred but for the defendant’s conduct. In the deepfake case, the answer is typically “yes” with respect to any single actor in isolation—the harm could plausibly have been produced by some other model, some other platform, some other prompter—and the test thus exonerates each defendant individually while collectively the harm has clearly been caused. The Chinese model has responded to this difficulty through codified personality rights with strict-liability platform obligations,^106^ a structural choice that resolves the pressure by abandoning the singular-tortfeasor presupposition.

### 7.5 The causation gap stated

The third gap is distinct from the second, though they often appear together in the same factual matrix. The second gap concerns the *number* of tortfeasors; the third gap concerns the *traceability* of causation across them. Even if Saudi or Jordanian doctrine successfully assigned primary liability to multiple defendants—a step neither has yet taken—the apportionment of responsibility across them would still face the evidentiary and analytical pressures identified in this section.^107^

The doctrinal solutions for the two gaps differ. For the second gap, clarification of Saudi Articles 132-134 and cautious extension of Jordanian Article 291 to algorithmic systems (and a reorientation of Jordanian application of Article 291) provides a workable path. For the third gap, the classical causation doctrine cannot itself bear the weight; it must be supplemented either by regulatory frameworks of the EU type,^108^ by burden-shifting evidentiary rules, or by both.^109^ The institutional preconditions for either supplement are non-trivial,^110^ and section 10 below distinguishes between the doctrinal and procedural reforms required.^111^

## 8 Comparative AI-governance frameworks: EU, US, UAE, and China

The doctrinal gaps identified in sections 5 through 7 are not unique to Saudi and Jordanian law. Most jurisdictions confronting deepfake harm in 2026 face a structurally similar problem: civil-liability frameworks shaped in the singular-tortfeasor era now strain against the multi-causal architecture of synthetic-media injury. The question is which jurisdictions have responded most coherently, and which of their responses are most plausibly transplantable into the Saudi-Jordanian context.^112^ I treat three primary comparators in this section: the European Union, the United States, and the United Arab Emirates as a regional intermediate model. The Chinese model, briefly engaged in section 7.4 above, is revisited at the end as a structurally distinct fourth archetype.^113^

### 8.1 The European Union: layered regulatory architecture

The EU response to deepfake harm operates through three intersecting instruments rather than a single regulation. The Artificial Intelligence Act of 2024 [Regulation (EU) 2024/1689, 2024], the Digital Services Act of 2022 [Regulation (EU) 2022/2065], and the General Data Protection Regulation of 2016 [Regulation (EU) 2016/679] each contribute a different layer to the overall framework. None alone is sufficient. Together they constitute the most structurally complete response any jurisdiction has yet produced.^114^

The AI Act’s contribution operates principally through Article 50, which becomes effective on 2 August 2026. Article 50(2) requires providers of generative AI systems to “ensure that the outputs of the AI system are marked in a machine-readable format and detectable as artificially generated or manipulated.”^115^ Article 50(4) imposes a parallel duty on deployers to disclose that any deepfake content “has been artificially generated or manipulated.”^116^ The first draft Code of Practice on Transparency of AI-Generated Content, published by the European Commission on 17 December 2025, operationalises these obligations through a multilayered marking approach combining metadata embedding, watermarking, and visible labelling.^117^

The DSA’s contribution is more structural. Articles 14 and 16 impose general transparency and notice-and-action obligations on all hosting services.^118^ Article 35, applicable to very large online platforms, requires “reasonable, proportionate and effective mitigation measures” tailored to identified systemic risks, with synthetic-media disinformation expressly within scope.^119^ This is a regulatory rather than tort response to the multi-causal causation problem identified in section 7: the platform’s liability is not contingent on classical causation analysis but on regulatory failure to mitigate risk.

The GDPR’s contribution has crystallised in the recent CJEU jurisprudence. The Russmedia judgment of 2 December 2025 held that an online-marketplace operator can be a controller under Article 4(7) of the GDPR for personal data contained in user-generated advertisements, with consequent liability under Article 82.^120^ Article 82 grants any person who has suffered material or non-material damage a right to compensation from the controller for damage caused by infringement of the regulation.^121^ Earlier jurisprudence—particularly Österreichische Post in 2023—established that mere infringement does not in itself ground compensation; actual non-material damage must be shown, but no *de minimis* threshold applies.^122^

A fourth EU-aligned development bears mention. Denmark in 2025 announced legislation extending a copyright-style right of reproduction (Tech Policy Press, 2025) to a person’s likeness, including AI-generated synthetic representations. If enacted, the Danish proposal would represent a structurally distinct response: a personality-rights extension rather than a regulatory or tort response.^123^^,^^124^

The architectural feature that distinguishes the EU framework is layering. Each instrument contributes a different mechanism—transparency, mitigation, individual remedy—without displacing the others.^125^ For a system facing the deepfake problem with a fragmented set of doctrinal tools, this layered architecture is more attractive than a single-statute response.

### 8.2 The United States: sectoral patchwork

The US response stands at the opposite pole. There is no comprehensive federal civil-liability framework for deepfake harm. The traditional immunity of platforms under Section 230 of the Communications Decency Act (1996) has shielded most synthetic-media intermediaries from direct civil liability, although this immunity is now eroding at its edges, particularly where platforms function as active amplifiers rather than passive hosts.^126^

Two federal instruments emerged in 2025–2026. The TAKE IT DOWN Act (2025), signed into law on 19 May 2025 as Public Law 119–12, criminalises the publication of non-consensual intimate visual depictions and digital forgeries and amends the Communications Act of 1934. Its notice-and-removal obligations on covered platforms require removal within 48 h of a valid request. Critically, the Act does not create a general private civil cause of action for damages; enforcement of the platform-removal obligations is assigned principally to the Federal Trade Commission. This is a regulatory-criminal response, not a civil-tort response.

The Defiance Act (2025) would do what the TAKE IT DOWN Act does not: create a federal civil cause of action for victims of non-consensual intimate digital forgeries. The Senate passed S. 1837 by unanimous consent in January 2026, but House passage remained pending at the time of writing. If enacted, the Act would represent a federal civil remedy specifically targeting one sectoral subset of deepfake harm, leaving corporate, political, and broader reputational deepfake harms to state-level law and existing tort doctrines.

At the state level, the picture is fragmented. Tennessee’s ELVIS Act of 2024 (Ensuring Likeness Voice and Image Security Act, 2024) was the first state statute to extend right-of-publicity protection expressly to “voice” and to AI-generated likenesses; California, Texas, and New York have followed with structurally divergent statutes.^127^^,^^128^^,^^129^

The architectural feature distinguishing the US framework—and the source of much of its inadequacy—is sectoral fragmentation. There is no single coherent civil-liability response to the deepfake problem as a category. For a civilian-Islamic system seeking comparative guidance, the US sectoral model offers limited transferable structure (Traverse Legal, 2025; Wiley LLP, 2025).

### 8.3 The United Arab Emirates: regional intermediate model

The UAE occupies an instructive intermediate position. Three features make the UAE comparison particularly valuable.

First, the Civil Code (United Arab Emirates) (1985), Article 316, provides the closest existing regional analogue to Jordanian Article 291. The provision’s textual proximity to Jordanian Article 291 is not coincidental; both descend through the Egyptian Civil Code of 1948 from the French garde tradition.^130^^,^^131^

Second, the UAE has paired this civilian foundation with two recent Western-influenced regulatory instruments: the Federal Decree-Law on Combating Rumours and Cybercrimes (UAE) (2021) and the Federal Personal Data Protection Law (UAE) (2021). The UAE thus operates a layered architecture analogous in form, though not in substantive depth, to the EU model.^132^

Third, the UAE has not enacted a comprehensive AI-specific statute analogous to the EU AI Act.^133^ This is a regulatory gap that mirrors the Saudi and Jordanian positions, and one of the lessons of the UAE comparison is that civilian-Islamic systems can address synthetic-media harm through their existing civil codes—where their existing custody-based provisions can be interpreted or clarified to cover high-risk algorithmic systems—without first needing to enact AI-specific legislation.

The empirical record reinforces the analytical point. The Gulf-region exposure to deepfake harm—most concentrated in the UAE and Saudi Arabia—has produced rising fraud volumes alongside the regulatory arrangements just described.^134^ The UAE has not yet been overwhelmed by the inadequacy of its existing civil-code framework, partly because Article 316 absorbs much of the layered-tortfeasor problem that Saudi law does not comfortably accommodate.

### 8.4 Comparative calibration

The comparative material supports a calibrated rather than transplant-based approach. The EU framework is the most useful because it combines synthetic-media transparency, platform-risk management, and data-protection remedies in a systemic structure. The US model is valuable for identifying concrete harm categories and First Amendment limits, but its sectoral design is less easily transposed into Saudi or Jordanian private law. The UAE model offers regional compatibility, while the Chinese model highlights how personality-rights protection and intermediary duties may be combined in a more interventionist framework.

For Saudi and Jordanian law, the useful lesson is not that AI regulation should replace civil liability. It is that civil liability needs regulatory support where deepfake harm depends on information that victims cannot access: training choices, watermarking policies, platform moderation logs, recommendation-system behaviour, and the chain of re-uploading. Transparency and burden-shifting are therefore not foreign additions to tort law; they are procedural conditions for making tort law practically enforceable in AI-mediated environments.

On institutional capacity, the comparison is more mixed. The EU framework presupposes a regulatory authority capable of risk-assessment evaluation. Saudi Arabia’s CST and SDAIA possess analogous (though more nascent) capacity; Jordan’s Telecommunications Regulatory Commission has a more limited mandate.^135^

On calibration to the specific gaps: for the first gap, all three comparators offer guidance. For the second gap, the UAE Article 316 provides the closest doctrinal analogue, with the EU’s DSA Article 35 offering a regulatory complement.^136^ For the third gap, the EU’s combination of risk-based regulatory obligations and Russmedia-style controllership extension offers the most calibrated response.^137^

The Chinese model, briefly, deserves a word. Functionally, the Chinese framework, anchored in the Civil Code (China) (2021), abolishes the singular-tortfeasor presupposition by codifying multiple recognised personality interests and by holding network platforms strictly liable. The institutional preconditions of this approach are particular to the Chinese regulatory structure. The Chinese model is functionally instructive but doctrinally and institutionally less transplantable into the Saudi-Jordanian context than the EU framework.^138^^,^^139^^,^^140^

The conclusion of this comparative survey is therefore directional rather than wholesale. The Saudi-Jordanian framework can most plausibly draw on the UAE’s Civil Code Article 316, together with Saudi Articles 132-134 and Jordanian Article 291, for the custody-based mechanism relevant to the second gap, and on selective elements of the Chinese strict-liability platform regime for the burden-shifting problem of the third gap.^141^ This is not legal transplant in the unqualified sense; it is calibrated borrowing (Table 2).^142^

**Table 2:** Comparative best-practice functions for the proposed model.

Jurisdiction/framework	Best-practice function	Use in this article
European Union	Defines deepfake legally, combines transparency duties, platform-risk obligations, and data-protection compensation.	Provides the principal layered governance reference for transparency, systemic risk, and evidential asymmetry.
United States	Offers sectoral responses to intimate-image abuse, publicity rights, and platform-immunity boundaries.	Illustrates the limits of fragmented regulation and the need for a more coherent private-law model.
United Arab Emirates	Retains a regional custody-of-things analogue through Civil Code Article 316 while using cybercrime and data-protection legislation.	Supplies a culturally and doctrinally closer bridge for adapting custody-based responsibility to algorithmic systems.
China	Combines personality-rights protection with stronger intermediary obligations.	Serves as a functional reference for platform responsibility, while remaining less directly transplantable institutionally.
Saudi Arabia and Jordan	Contain moral-harm, causation, and custody-related doctrines but lack a fully operational deepfake-liability model.	Function as the receiving frameworks for the proposed layered civil-liability reform.

Two methodological observations close this section. First, the EU framework’s administrative-regulatory mechanisms are more readily transplantable than its civil-procedural mechanisms.^143^ Second, the empirical record provides functional grounds for the same calibration.^144^

A final caveat. The comparative exercise just undertaken does not yet ask whether the Sharī’ah foundation that underlies both Saudi and Jordanian civil law generates its own normative resources for synthetic-media harm.^145^ This question is the subject of section 9.^146^ The full reform package is then articulated in section 10.^147^^,^^148^

## 9 The Sharī’ah foundation and the limits of doctrinal borrowing

The reform proposals advanced in section 10 below draw heavily on EU and UAE comparative material. Before turning to those proposals, this short section addresses a question that any responsible reform programme for a Sharī’a-grounded legal system must confront: do the deeper normative resources of the Sharī’ah itself bear on the deepfake problem, and if so, what role do they play in the reform argument?

I argue here that the Sharī’ah foundation generates a substantial normative resource for synthetic-media harm—principally through *ḥifẓ al-’irḍ* (the preservation of personal honour) and the prohibition of *buhtān* (calumny)—but that this resource operates as interpretive guidance rather than as operative doctrine. The reform proposals of section 10 draw their statutory mechanism from the comparative material; the Sharī’ah foundation supplies their normative anchoring.

### 9.1 Ḥifẓ al-’irḍ in the Maqāṣid tradition

The classical articulation of maqāṣid al-sharī’a (al-Shāṭibī, 1997; Ibn ‘Āshūr, 2001; al-Raysūnī, 1995; al-Qaraḍāwī, 1990) identifies five necessary objectives (al-ḍarūriyyāt al-khams): the preservation of religion, life, intellect, lineage, and property. A sixth—ḥifẓ al-’irḍ, the preservation of personal honour and dignity—has been recognised by classical jurists either as an independent objective or as subsumed under the preservation of lineage. The contemporary literature increasingly treats ḥifẓ al-’irḍ as autonomously articulated, particularly in contexts of digital harm.^149^^,^^150^

The scriptural anchoring is robust. The Qurʾān prescribes a ḥadd punishment for false accusation of unchastity (qadhf) in Sūrat al-Nūr 24:4, and forbids defamatory naming, suspicion, spying, and backbiting in 49:11–12. Classical exegesis (al-Qurṭubī, 2006; Ibn Qudāma, 1997; al-Nawawī, 1998; al-Qaraḍāwī, 1991) treats qadhf as the foundational scriptural anchor of ḥifẓ al-’irḍ. The category of buhtān (Ibn Manẓūr, 1414AH)—broader than qadhf—encompasses the attribution to another of any falsehood capable of damaging reputation (Awda, 1992). The deepfake—by its very structure—fits within buhtān more precisely than within any narrower defamation category, because it generates falsehood as an artefact of its construction.^151^^,^^152^^,^^153^

The functional resemblance between *ḥifẓ al-’irḍ* and the European personality-rights tradition is substantial but not identical.^154^ The reform argument therefore cannot present the European model as merely a vehicle for what *ḥifẓ al-’irḍ* already requires; it must present the European model as a calibrated mechanism that the Sharī’ah foundation justifies but does not dictate.

### 9.2 The limits of Maqāṣid-level reasoning

A categorical distinction is operative throughout the uṣūl al-fiqh tradition (al-Zuḥaylī, 1986; al-Subkī, 1424AH; al-Suyūṭī, 1990; al-Qarāfī, 1998): maqāṣid are guiding principles (mabādiʾ tawjīhiyya), not operative legal rules (aḥkām ‘amaliyya). They direct interpretation; they do not, of themselves, generate enforceable doctrine. The transformation of maqāṣid-level reasoning into operative legal rules typically requires either codified statute or—in the Sharī’ah-only frame—the issuance of al-fatāwā al-jamā’iyya (collective fatwa). As of 2026, no such collective fatwa specifically addresses civil liability for deepfake-induced moral harm.^155^^,^^156^

The implication for the reform argument is precise. The *ḥifẓ al-’irḍ*-grounded normative claim supports the doctrinal direction proposed in section 10, but does not by itself supply the operational text.^157^ The contemporary scholarly literature on digital *ḥifẓ al-’irḍ* treats the principle as ethically operative but properly stops short of claiming codified status.^158^

Two further uṣūl-level principles support the direction of reform. The first is darʾ al-mafāsid muqaddam ‘alā jalb al-maṣāliḥ (the prevention of harm takes precedence over the procurement of benefit) (Abū Ṣa’d, 2010), which applies with particular force in the synthetic-media context. The second is the doctrinal caution against using maqāṣid as autonomous source of legal rules—a caution articulated most clearly in the uṣūlī literature.^159^^,^^160^

### 9.3 Implications for the reform programme

Three operational implications follow. First, the proposals are calibrated to operate within the existing constitutional architecture of both Saudi Arabia and Jordan.^161^ The Sharī’ah foundation supports the amendments without supplanting their statutory mechanism.^162^

Second, the foundation strengthens the interpretive case for resolving textual ambiguities in favour of broader protection. The Saudi Civil Transactions Law’s grounding in the Ḥanbalī tradition leaves interpretive room for *maqāṣid*-supported readings of ambiguous provisions;^163^ the Jordanian Civil Code’s broader doctrinal openness to *maqāṣid*-style reasoning is likewise hospitable.^164^ Specific applications follow.^165^

Third, the foundation conditions the reception of foreign doctrinal models. The reform proposals draw on EU and UAE comparative material, but the comparative argument operates within a Sharī’ah-grounded constraint: the imported elements must be evaluable against, and consistent with, *ḥifẓ al-’irḍ* and the *dar’ al-mafāsid* principle.^166^ The Sharī’ah foundation does not dictate a particular external model; it constrains the field of legitimate options.^167^ The EU framework selected as principal template in section 8 satisfies this constraint with particular clarity.^168^

## 10 Towards a layered liability reform model

The preceding analysis points towards a layered reform model. The model does not impose strict liability on every participant in the deepfake ecosystem. Nor does it leave the victim to sue only the anonymous human uploader. It allocates responsibility by function: those who design or deploy generative AI systems should bear duties connected to foreseeable misuse and transparency; those who host and amplify synthetic media should bear duties connected to notice, risk mitigation, and algorithmic amplification; and those who create, upload, or redistribute harmful deepfakes should remain directly liable for intentional or negligent misuse.

The proposed reforms preserve the legal core of Saudi and Jordanian civil liability while making that core operational in an AI environment. They are organised around the three gaps diagnosed above: claimant protection, layered actors, and algorithmic causation.

### 10.1 Closing the first gap: amending Article 138(2)

The natural-person limitation in Saudi Article 138(2) requires textual amendment.^169^ I propose the following revised text:

**Proposed Saudi Civil Transactions Law, Article 138(2) [revised].**

Moral harm includes the physical or psychological suffering inflicted on a natural or juridical person as a result of injury to body, freedom, honour, reputation, or social standing, and includes—in the case of the juridical person—injury to its commercial reputation or its reputational capital.^170^

The amendment introduces three changes. First, it extends the protected category from “natural person” to “natural or juridical person.” Second, it preserves the existing enumerated heads of harm. Third, it introduces a calibrated head specific to juridical persons: “commercial reputation” (*al-sum’a al-tijāriyya*) and “reputational capital” (*ra’smāl i’tibārī*).^171^ The drafting precedent in French Civil Code Article 1240, Egyptian Article 222, and UAE Article 290 supports the structural choice.^172^

The Jordanian counterpart problem requires no statutory amendment but judicial reorientation. Article 267 of the Jordanian Civil Code is textually adequate; the restrictive judicial gloss anchored to subjective psychological experience is not compelled by the text and may be reversed by the Court of Cassation through a guiding decision.^173^

### 10.2 Closing the second gap: clarifying Saudi Article 132 and Jordanian Article 291

The second gap does not require creating a custody rule from nothing in Saudi law. Saudi Article 132 already imposes liability on the custodian of a thing requiring special care, and Article 134 defines custody by actual authority. The reform required is clarification that these provisions may apply, where appropriate, to high-risk algorithmic systems and generative-AI tools when the defendant exercises effective control.^174^

**Proposed Saudi Civil Transactions Law, Article 124 bis [new].**

Proposed clarification to Saudi Article 132/134: For the purposes of Articles 132 and 134, high-risk algorithmic systems and generative-AI tools may be treated as things requiring special care where their operation creates foreseeable risks of reputational, identity-based, or non-material harm, and where the defendant exercises actual authority over design, deployment, or distribution.^175^

The amendment imports the Jordanian-Egyptian-French custody-of-things doctrine into the Saudi framework, with one calibrated technical specification: the express inclusion of “technical and generative-algorithmic systems” within the article’s scope.^176^ This specification serves two functions. It signals to courts that the article reaches generative AI systems without restricting its general applicability to other categories of harm-generating things. And it provides interpretive support for treating both the foundation-model developer and the platform operator as custodians of distinct risk-generating systems within the meaning of the article.

The Jordanian counterpart requires no amendment but exegetical extension. Article 291, as drafted, already accommodates the algorithmic-system context; Jordanian Article 291 through cautious extension to algorithmic systems, and Saudi Articles 132-134 through clarification of effective control over high-risk generative systems.

### 10.3 Strengthening the data-protection framework

The third structural reform addresses the Russmedia-style controllership extension: the introduction into both the Saudi and Jordanian PDPLs of a private cause of action analogous to GDPR Article 82.^177^ I propose the following addition to the Saudi PDPL:

**Proposed Saudi PDPL, new Article 30 bis.**

Any person who has suffered material or non-material damage as a result of an infringement of this Law shall have the right to receive compensation from the controller or processor for the damage suffered. Compensation under this Article is in addition to, and not in substitution for, any administrative or criminal sanctions imposed under the Implementing Regulations.

The Jordanian PDPL of 2023 requires a parallel amendment, with calibration to the existing Jordanian civil-procedure framework.^178^

### 10.4 Closing the third gap: burden-shifting in algorithmic causation

The causation-opacity problem cannot be resolved through substantive amendment alone. It requires a procedural rule shifting the burden of proof in defined circumstances. I propose the following insertion into the Code of Civil Procedure (Saudi Arabia) (2013) (and a parallel amendment to the Code of Civil Procedure (Jordan) (1988)):^179^

**Proposed Saudi Code of Civil Procedure, new Article 104 bis.**

Where the plaintiff establishes by a preponderance of probability that harm has resulted from the operation of a generative algorithmic system causally connected to the defendant, the burden of proof shifts to the defendant in respect of the absence of causation or the adoption of appropriate technical safeguards.^180^

The “preponderance of probability” (*al-iḥtimāl al-rājiḥ*) threshold is the standard doctrinal formulation in Saudi and Jordanian civil procedure for prima facie showings.^181^ The rule is structurally analogous to res ipsa loquitur in common-law systems and to the French *présomption de responsabilité*.

### 10.5 Supplementary administrative regulation

Beyond the civil-code and PDPL amendments, the reform package contemplates a supplementary administrative regulation issued under the existing rule-making authority of the Saudi CST and SDAIA, modelled on AI Act Article 50(2)–(4) and the December 2025 draft Code of Practice (European Commission, 2025). The regulation should require (a) machine-readable marking of AI-generated content; (b) visible labelling of deepfakes for public-interest content; (c) systemic-risk assessment by very large platforms operating in the Saudi market; and (d) cooperation with the regulatory authority on takedown and remediation.^182^^,^^183^

### 10.6 Phasing and internal coherence

The reform package contains five elements: (a) Saudi Article 138(2) amendment plus Jordanian judicial reorientation of art 267; (b) clarification of Saudi Articles 132-134 plus targeted Jordanian exegesis of Article 291; (c) Saudi and Jordanian PDPL amendments; (d) supplementary administrative regulation; and (e) procedural burden-shifting rule. Items (c) and (d) can proceed in parallel under existing rule-making authority. Items (a), (b), and (e) require legislative process.^184^

The internal architecture is complementary. The civil-code amendments establish substantive grounds of liability; the data-protection amendment provides a parallel private-right-of-action track; the administrative regulation supplies the technical infrastructure; and the burden-shifting rule operationalises the substantive provisions in the algorithmic-causation contexts.^185^ The Jordanian reform sequence is functionally parallel.^186^

Implementation challenges remain.^187^ None is insurmountable, but each requires attention as the reforms move from textual amendment to operational deployment. The reform proposals are offered as a coherent package; partial adoption is possible but will produce uneven coverage of the gap structure.^188^

## 11 Discussion: why the problem persists

The persistence of deepfake harm is not simply a problem of legislative delay. It reflects a mismatch between the architecture of the harm and the architecture of the available legal tools. Civil codes tend to ask who caused the harm; platform regulation tends to ask who should reduce systemic risk; data-protection law tends to ask whether personal data was processed unlawfully. Deepfake injury often engages all three questions at the same time.

This is why a layered model is preferable to a single doctrinal solution. A transparency duty may help identify artificial content, but it does not compensate the victim. A data-protection remedy may address unlawful processing, but it may not fully capture reputational or identity-based harm. A classical tort claim may identify a wrongdoer, but it may fail where the decisive evidence is held by platforms or developers. The problem therefore persists because each legal tool sees only part of the chain.

The proposed model also requires ethical calibration. Civil-liability reform should not impose open-ended platform liability, suppress lawful expression, or discourage legitimate innovation in generative AI. The better approach is proportionate responsibility: liability and evidential burdens should follow control, foreseeability, and access to relevant technical information. This preserves space for innovation while ensuring that victims of serious synthetic-media harm are not left without an effective remedy.

## 12 Conclusion and future research

This article set out to answer a practical private-law question: how should civil-liability doctrine allocate responsibility when deepfake-induced harm is generated, amplified, and redistributed through a chain of actors rather than through a single wrongdoer? The answer offered here is that deepfake harm should be treated as a layered AI-accountability problem with a civil-liability core. Saudi and Jordanian law already contain concepts capable of doing part of the necessary work - general civil wrong, moral harm, direct and indirect causation, custody of things in Jordan, and Sharī’ah-sensitive protection of honour and reputation. The difficulty is that these concepts were not designed for distributed technical and platform architectures.

The analysis identified three structural gaps. The first is a claimant-protection gap: Saudi Article 138(2) restricts moral-harm protection to natural persons, and Jordanian practice may narrow Article 267 by tying moral harm to subjective psychological suffering. The second is an actor-attribution gap: deepfake harm is created through several layers, while classical liability doctrine still tends to search for a single wrongdoer. The third is a causation-evidence gap: victims often cannot access the technical and platform evidence needed to prove how the harm was generated and amplified.

The comparative analysis supports a calibrated reform strategy. The EU AI Act, the Digital Services Act, and GDPR-based remedies provide the most coherent external guidance because they combine transparency, platform responsibility, and remedial logic. The US model is important but more fragmented. UAE and Chinese materials are useful as additional reference points, though each requires careful adjustment before any local use.

The article’s main contribution is a targeted reform model rather than a call for wholesale replacement of existing doctrine. It proposes clearer protection for juridical-person reputation where appropriate; a Saudi custody-based rule for algorithmic systems; controlled burden shifting in algorithmic-causation cases; stronger private-law links to data-protection regimes; and proportionate transparency obligations for synthetic-media systems. These reforms do not require abandoning the civil-law or Sharī’ah-sensitive structure of Saudi and Jordanian law. They require that structure to be made operational in a new technological setting.

The broader conclusion is that civil liability and AI governance should be treated as mutually reinforcing fields. Deepfake harm cannot be remedied by technical labelling alone, and it cannot be handled by classical tort doctrine without adjustment. A workable response must preserve the discipline of private law while recognising the realities of generative AI, platform amplification, and digital identity.

### 12.1 Future research

Future research should move in four directions. First, once Saudi and Jordanian courts begin to decide deepfake-related civil cases, the layered model proposed here should be tested against actual litigation patterns. Secondly, further work is needed on platform evidence, including moderation logs, recommender-system records, watermarking failures, and disclosure duties. Thirdly, comparative research should examine whether data-protection remedies can realistically carry the weight of non-material and reputational harm in Arab civil-law systems. Fourthly, future scholarship should develop procedural rules for expert evidence and burden shifting in algorithmic-causation disputes.

## Footnotes

1 I distinguish four strands of contemporary literature: (a) US-centred deepfake-tort scholarship; (b) European AI-and-data-protection scholarship; (c) Asian comparative scholarship; and (d) Sharī’a-grounded scholarship on digital harm. Each is engaged in turn.

2 Chesney and Citron (2019) is the foundational treatment, identifying the four-dimensional threat (privacy, democracy, national security, market integrity) and proposing the “virality index” framework for damages calibration.

3 Citron and Solove (2022) extends the analysis to the broader category of privacy harms and develops the evidentiary-asymmetry argument referenced at section 7.3 above.

4 Kadri and West (2025) provides the most comprehensive recent state-by-state survey, identifying both the common-law tort frameworks operating intermittently and the emerging statutory responses (publicity-rights extensions, election-integrity statutes, intimate-imagery protections).

5 Delfino (2024) addresses the courtroom-evidentiary dimension—the “deepfake defense” problem in which any video evidence may be challenged as potentially synthetic—extending the analysis beyond civil-tort liability into criminal procedure.

6 See further the surveys in Wiley LLP (2025) and Institute for Law & AI (2026).

7 On the EU AI Act’s genesis and structure, see Edwards (2024) and Bradford (2023), Digital Empires, ch. 6.

8 On the DSA, see Wilman (2022); and on the GDPR’s art 82 jurisprudence, the recent commentaries cited above.

9 The civilian-doctrinal commentary on AI liability is led by Bacache (2020) and Loiseau (2019) in France; for the broader European-comparative frame, see Sebastian Lohsse, Reiner Schulze and Dirk Staudenmayer (eds), Liability for Artificial Intelligence and the Internet of Things (Nomos/Hart 2019).

10 The Russmedia framework is the most recent and most consequential development; preliminary academic commentary appeared in early 2026.

11 The Sharī’a-grounded literature is sparse but emerging. The principal contributions include Khotimah (2024), Uriawan et al. (2025), and Bawono et al. (2025).

12 These works, while methodologically valuable, treat ḥifẓ al-’irḍ as ethical principle rather than as the foundation of a specific civil-liability proposal. The present article extends their approach by linking the Sharī’a foundation explicitly to statutory-reform proposals.

13 The Arab civilian-doctrinal literature on deepfake liability is, at the time of writing, even more limited. Articles addressing AI civil liability more broadly include the author’s prior work on AI autonomous-decision liability (under journal review) and a small number of comparative pieces published in regional law reviews.

14 No published English-language article, to the best of my knowledge, has examined the Civil Transactions Law (Saudi Arabia) (2023) against deepfake-induced harm with the specific focus on Article 138(2) developed at section 5 below.

15 The Jordanian-specific literature is similarly thin; the principal Arabic-language treatments are al-Zu’bī (2018) for the general civil-liability framework and Sulṭān (2018) for the obligations doctrine.

16 On the Sanhūrian synthesis and its transmission to other Arab civil codes, see al-Sanhūrī (1952), introduction. For a contemporary assessment, see Mallat (2007), ch. 7.

17 Civil Transactions Law, Royal Decree M/191 of 18/11/1444 AH (corresponding to 6 June 2023), published in Umm al-Qurā Official Gazette No 4995. The law entered into force on 16 December 2023, 180 days after publication. For an early commentary, see Al-Ḥaqbānī (2024) (in Arabic, on file with author).

18 On the doctrinal continuities between classical Islamic jurisprudence and the modern Arab civilian codes, see Abū Zahra (1977); and more recently, Hallaq (2009), ch. 16. The mubāshir-mutasabbib distinction itself originates with the Ḥanafī jurists; see Ibn Nujaym (1983), pp. 169–180.

19 Civil Transactions Law (Saudi Arabia, 2023) arts 119–145.

20 Compare French Civil Code, art 1240 (formerly art 1382): “Tout fait quelconque de l’homme, qui cause à autrui un dommage, oblige celui par la faute duquel il est arrivé, à le réparer.” Although the textual formulations are similar, the underlying conceptual frameworks differ: the Saudi provision rests on the Ḥanbalī concept of ḍamān (guarantee/liability) rather than the Romance faute. See al-Mu’ajjal (1442AH), pp. 178–185.

21 Civil Transactions Law (Saudi Arabia, 2023) art 121. The provision establishes a rebuttable causation presumption where the harmful act is attributed to its actor. Translation by author.

22 On the historical pedigree of the distinction in Ḥanafī jurisprudence, see al-Qarāfī (1998), vol. 4, pp. 26–32. For its codified afterlife in twentieth-century Arab civilian codes, see Abū Ṣa’d (2010), pp. 56–92.

23 Civil Transactions Law (Saudi Arabia, 2023) arts 122 (animal-keeper liability), 123 (vicarious liability for the act of a subordinate), and 124 (responsibility for buildings in disrepair).

24 The codification of moral harm in Article 138 ended decades of doctrinal uncertainty in Saudi jurisprudence regarding the recoverability of non-pecuniary damages outside specific penal contexts. See al-Sanhūrī (1952) 911–925; and the discussion in Al-Ḥaqbānī (2024).

25 Civil Transactions Law (Saudi Arabia, 2023) art 138(2): “Moral harm includes the physical or psychological suffering inflicted on the natural person (al-shakhṣ dhū al-ṣifa al-ṭabī’iyya) as a result of injury to his body, freedom, honour, reputation, or social standing.” Translation by author.

26 Civil Transactions Law (Saudi Arabia, 2023) art 138(3) (heritability) and art 138(4) (judicial discretion in quantum). On the operation of judicial discretion in moral-harm quantification under Saudi law, see Al-Ḥaqbānī (2024).

27 The Egyptian Civil Code, art 222, and the Jordanian Civil Code, art 267, both contain moral-harm provisions without comparable natural-person restrictions. The Saudi codifiers’ deliberate departure from these models is treated more fully in section 5 below.

28 Personal Data Protection Law, Royal Decree M/19 of 9/2/1443 AH, fully effective 14 September 2023; see also the Implementing Regulations issued by the Saudi Data and AI Authority (SDAIA), 2023. The PDPL provides administrative and criminal enforcement mechanisms but does not itself create a private right of action for moral damages independent of the Civil Transactions Law’s general framework.

29 Jordanian Civil Code (Law No 43 of 1976), arts 256–292, published in the Official Gazette of the Hashemite Kingdom of Jordan No 2645 of 1 August 1976.

30 Civil Code (Jordan) (1976) art 256. Translation by author.

31 Civil Code (Jordan) (1976) arts 257–266. For doctrinal commentary, see al-Zu’bī (2018), pp. 138–274; and Sulṭān (2018).

32 Civil Code (Jordan) (1976) art 267. The full text reads: “The right to indemnity covers moral harm as well; thus any infringement on the freedom, honour, reputation, social standing, or financial standing (al-i’tibār al-mālī) of another renders the perpetrator liable to indemnify.” Translation by author.

33 Civil Code (Jordan) (1976) art 291(1). Translation by author.

34 See Egyptian Civil Code (Egypt) (1948), art. 178; Cass ch réun, 13 February 1930 (Jand’heur), DP 1930.1.57, note Ripert. On the doctrinal genealogy connecting French garde to its Egyptian and Jordanian transpositions, see al-Sanhūrī (1952), p. 1075 ff; and Terré et al. (2018), nos 882–902.

35 Jordanian Cybercrimes Law (No 17 of 2023), in force from 13 September 2023, repealing Law No 27 of 2015; and Jordanian Personal Data Protection Law (No 24 of 2023), in force from 17 March 2024.

36 Jordanian law expressly codifies direct and indirect harm through Articles 257–258. Saudi Article 121 is narrower: it establishes a rebuttable causation presumption where the harmful act is attributed to its actor, rather than a full mubāshir-mutasabbib framework. See al-Sanhūrī (1952) 962–978; and the application of the framework in the case-law surveyed by al-Zu’bī (2018) 138–152.

37 Both the Saudi PDPL of 2021 and the Jordanian PDPL of 2023 draw closely on Regulation (EU) 2016/679 (General Data Protection Regulation) [2016] OJ L119/1 in their definitional architecture, processing principles, and data-subject rights. The conceptual transposition is most visible in the structure of consent, lawful bases, and cross-border transfer regimes. See further the comparative discussion in section 8 below.

38 On the Arup case—in which deepfake technology was used to impersonate the chief financial officer of the Hong Kong branch of the British engineering firm Arup, resulting in fraudulent transfers of approximately US$25 million in January 2024—see Bosher (2026).

39 Civil Transactions Law (Saudi Arabia, 2023) art 138(2). On the Saudi canons of construction generally, and the strict approach to enumeration in particular, see al-Mu’ajjal (1442AH), p. 188–202.

40 Article 138(2) of the Civil Transactions Law (Saudi Arabia, 2023). On the legislative-drafting principle that enumerated provisions are generally exhaustive within their domain (al-’add yaqḍī ‘alā l-iṭlāq), see Tāj al-Dīn al-Subkī, Jam’ al-jawāmi’ (‘Abd al-Mun’im Khalīl Ibrāhīm ed., Dār al-Kutub al-’Ilmiyya 1,424 AH) vol 2, 56.

41 On the canon “the specific qualifies the general” (al-khāṣṣ yuqayyid al-’āmm) and its application in modern Arab civilian doctrine, see al-Subkī (1424AH) vol 2, 56; and al-Mu’ajjal (1442AH), p. 192–198.

42 See al-Mu’ajjal (1442AH), p. 178–185, on the established interpretive hierarchy in Saudi statutory construction. The 2023 Code itself contains no general interpretive provision overriding this hierarchy.

43 Classical Ḥanbalī jurisprudence accepted ḍamān for harm done to waqf endowments and to the reputational interests of charitable institutions on a case-by-case basis, treating the entity’s collective interest as protected through the trustee. See Ibn Qudāma, al-Mughnī (Dār ‘Ālam al-Kutub 1997) vol 7, 405–422. The 2023 Code’s natural-person restriction therefore represents a narrowing of, rather than continuity with, this classical position.

44 Civil Code (Jordan) (1976) art 267; cf. Egyptian Civil Code (Jordan, 1976) art 222.

45 See al-Zu’bī (2018), p. 256–274 (surveying the Jordanian Court of Cassation’s case-law and noting the consistent anchoring of moral harm to subjective psychological experience); and Sulṭān (2018). The Jordanian construction parallels, but is not identical to, the more cautious Egyptian approach to corporate moral harm.

46 See Maḥmūd Jamāl al-Dīn Zakī, Mushkilāt al-masʾūliyya al-madaniyya [Problems of Civil Liability] (2nd edn, Maṭba’at Jāmi’at al-Qāhira 1990) vol 2, 145–167; and Rūwayda Mūsā, al-Ta’wīḍ ‘an al-ḍarar al-ma’nawī fī al-qānūn al-Filasṭīnī—dirāsa muqārana (Master’s thesis, Arab American University, Palestine, 2020).

47 Pound (1910). The methodological distinction Pound articulated remains operative in functional comparative law; see Zweigert and Kötz (1998), p. 36–42.

48 On the comparative-law lessons of jurisdictions that converge on outcomes from divergent texts, and the implications for legal-transplant theory, see Alan Watson, Legal Transplants: An Approach to Comparative Law (2nd edn, University of Georgia Press 1993) 21–30.

49 Bosher (2026). For the Hong Kong Police Force’s public statements on the Arup investigation, see Hong Kong Police Force (2024).

50 Sumsub (2024), Table 4.

51 World Economic Forum, Global Cybersecurity Outlook 2024 (WEF, January 2024) ch. 3.

52 See CST (2024); and the regional analysis in Arab News (2025), reporting Sumsub data on the 600 per cent year-on-year increase in deepfake incidents in the Kingdom in the first quarter of 2024.

53 On the concept of corporate reputational capital and its market implications, see Fombrun (1996); and for the digital-era extension, Diermeier (2011).

54 See Citron and Solove (2022), pp. 820–832; and Chesney and Citron (2019), pp. 1772–1779.

55 Case C-492/23, X v Russmedia Digital SRL and Inform Media Press SRL (2025) (CJEU, Grand Chamber, 2 December 2025). The Grand Chamber held that the operator of an online marketplace can be a controller within the meaning of art 4(7) of the GDPR for personal data contained in user-generated advertisements, with consequent liability under art 82, notwithstanding the e-Commerce Directive’s hosting safe harbour. The judgment’s significance for synthetic-media platform liability is treated in section 8.

56 Civil Transactions Law (Saudi Arabia, 2023) art 138(4).

57 The distinction between provisions establishing recoverability of a head of damage (eligibility) and provisions calibrating its measurement (quantum) is foundational in civilian liability doctrine. See al-Sanhūrī (1952), pp. 1110–1,118; for the modern French statement, Viney, Jourdain and Carval (2013), nos 245–260.

58 Civil Transactions Law (Saudi Arabia, 2023) art 29(1): “the exercise of a right shall not be abusive.”

59 On the doctrinal limits of ta’assuf fī isti’māl al-ḥaqq as a corrective rather than substantive ground of liability, see al-Sanhūrī (1952) 819–832; and on its modern Saudi application, al-Mu’ajjal (1442AH) 211–218.

60 Civil Transactions Law (Saudi Arabia, 2023) art 119. The general clause governs liability for material harm; the moral-harm head is exclusively governed by art 138.

61 On the French jurisprudence permitting moral damages to legal persons under former art 1,382 (now art 1,240) of the French Civil Code, see Cass com (2012), no. 11–10.278; for doctrinal analysis, Bacache (2020).

62 Personal Data Protection Law (Saudi Arabia, 2021), arts 32–37 (penalties and enforcement mechanisms). The PDPL’s enforcement architecture is administrative and criminal; private-law remedies for moral damages remain governed by the Civil Transactions Law’s general framework. The same conclusion holds for the Jordanian PDPL of 2023.

63 On methodological commitment to functional comparison as the proper frame for assessing such convergence, see Zweigert and Kötz (1998), p. 36–46.

64 Detailed legislative-reform proposals for both jurisdictions are set out in section 10. The textual amendment of Saudi art 138(2) is doctrinally less demanding than reversing accumulated Jordanian Court of Cassation case-law, but both reforms are necessary to close the gap identified in this section.

65 Watson (1993), p. 21–30, on the relative ease of textual amendment compared with judicial reorientation in transplant scenarios.

66 Civil Transactions Law (Saudi Arabia, 2023) art 119; Civil Code (Jordan) (1976) art 256. The grammatical singular (“man aḥdatha ḍararan”, “anyone who causes harm”) in both provisions is doctrinally significant: it instantiates a presupposition of singular agency at the level of the textual frame.

67 On the singular-actor presupposition in classical Islamic jurisprudence, see al-Qarāfī (1998) vol 4, 26–32, treating each al-mubāshir as the agent whose conduct is the proximate vehicle of the harm; and al-Sanhūrī (1952), p. 832–845, recording the modern civilian articulation of the same assumption. The presupposition was historically uncontroversial because the cases the doctrine addressed were structurally singular.

68 On the paradigmatic case-types—collisions, structural collapse, fraud, intentional injury—that shaped classical liability doctrine, see al-Sanhūrī (1952), p. 850–895; and Henri Mazeaud, Léon Mazeaud and Jean Mazeaud, Leçons de droit civil, t II vol 1: Obligations (F Chabas ed., 9th edn, Montchrestien 1998) nos 437 ff.

69 The four-layer architecture is functional rather than statutory; my taxonomy draws on and extends the typologies offered in Chesney and Citron (2019), p. 1761–1772; and Kadri and West (2025), pp. 528–540. For an alternative typology emphasizing intermediary obligations, see Peng and Lee (2025).

70 On the typical absence of specific awareness on the part of foundation-model developers regarding particular harmful uses, see Peter Henderson and others, “Foundation Models and Fair Use” (2023) preprint, arXiv:2303.15715, 7–12. The argument is methodological as much as doctrinal: the developer’s position resembles that of a manufacturer of dual-use goods more than that of a classical tortfeasor.

71 On training-data curation, output filtering, watermarking, and access controls as the principal levers through which developer choice shapes the universe of possible misuse, see EU AI Act, art 50(2); and Bradford (2023), ch. 6. The EU’s recent Code of Practice on Transparency of AI-Generated Content (first draft, 17 December 2025) operationalises these levers under a regulatory rather than tort framework.

72 On the prevalence of anonymity, jurisdictional unreachability, and judgment-proofness among individual deepfake creators, see Flynn et al. (2025); and DiResta (2024), ch. 4.

73 On the platform as active amplifier rather than passive host, see Gillespie (2014), p. 167; and Klonick (2018), pp. 1630–1,638. The recent litigation trend treating amplification as a distinct ground of liability is surveyed in Wiley LLP (2025).

74 On viral re-distribution as a structurally distinct causal layer, see Chesney and Citron (2019) 1773–1779 (proposing a “virality index” measure); and DiResta (2024) ch. 4. The four-layer model is offered as analytically distinct rather than as a closed set.

75 Civil Transactions Law (Saudi Arabia, 2023) art 121; cf. the parallel doctrinal operation in Jordanian law described above.

76 On the operation of art 121’s default rule in cases involving anonymous or judgment-proof direct causers, see al-Mu’ajjal (1442AH), p. 218–225. The classical sources do not directly address the modern problem of an anonymous mubāshir located outside the jurisdiction; the doctrine’s structural assumptions did not contemplate it.

77 On the classical treatment of inanimate causes (al-asbāb al-māddiyya), and the principle that causation is attributed to the closest agentic actor, see Ibn Nujaym (1983), p. 173–178; and Abū Ṣa’d (2010), p. 78–92. The principle was developed in cases involving such things as falling roof tiles and runaway carts; its application to algorithmically generated synthetic content is offered as an interpretive analogy rather than as direct doctrinal authority.

78 The Saudi Code does not expressly provide for joint primary liability of structurally distinct concurrent tortfeasors. Article 121 contemplates the concurrence of mubāshir and mutasabbib as types, not the concurrence of multiple mubāshirūn as independent primary actors. *Cf* the more developed treatment in Jordanian and Egyptian doctrine: al-Sanhūrī (1952), p. 932–945.

79 Civil Code (Jordan) (1976) art 291(1) (full text quoted at section 4.3 above).

80 On the traditional sphere of art 291’s application—vehicles, machinery, animals, structurally hazardous objects—see al-Zu’bī (2018), p. 296–318; and Muḥammad Waḥīd al-Dīn Suwwār, Sharḥ al-qānūn al-madanī al-Urdunī—maṣādir al-ḥuqūq al-shakhṣiyya (Maktabat Dār al-Thaqāfa 1996) 489 ff.

81 Arrêt Jand’heur; and Cass ch réun, 2 December 1941 (Franck), DC 1942.25, note Ripert (settling the modern formulation of garde as comprising usage, direction and contrôle). For the modern doctrinal exposition, see Terré et al. (2018) nos 882–902.

82 On the conceptual extension of garde to algorithmic and software contexts, see Bacache (2020) and Loiseau (2019). Both authors note that French doctrine has not yet definitively endorsed the extension; the doctrinal possibility, however, is real.

83 Based on systematic review of the published case-law databases of the Jordanian Judicial Council, the Jordanian Bar Association, and the Qistas database, conducted from 2020 through Q1 2026, using keywords al-tazyīf al-’amīq [deepfake], synthetic media, and artificial intelligence in conjunction with ta’wīḍ [indemnity] and ḍarar adabī [moral harm]. The review disclosed no published cases applying art 291 to a generative-AI system or platform algorithm. The methodological details are set out in section 5 (research method).

84 On the French academic articulation of garde de l’algorithme, see Bacache (2020), Loiseau (2019), and the cautious doctrinal commentary in Viney, Jourdain and Carval (2013), nos 685 ff.

85 Civil Transactions Law (Saudi Arabia, 2023) arts 122 and 124. Saudi commentary has thus far treated these as closed enumerations; see al-Mu’ajjal (1442AH), p. 226–234.

86 An expansive reading of art 119 (aḥdatha = “caused” or “brought about”) to encompass the platform and developer as concurrent perpetrators of the eventual harm is doctrinally available but leaves apportionment unguided. The Code provides no rule for the apportionment of damages among concurrent primary tortfeasors of distinct structural roles. See Al-Ḥaqbānī (2024).

87 The analogical extension of art 122 (animal-keeper liability) to algorithmic systems would be doctrinally awkward. Animals are agentic creatures whose harm is foreseeable from the keeper’s position; foundation models are non-agentic computational systems whose harm is foreseeable only at the category level. The two are not structurally equivalent.

88 On Saudi doctrinal caution regarding analogical extension within statutory interpretation, particularly in matters of liability (ḍamān), see al-Mu’ajjal (1442AH), p. 245–256. The Saudi Code’s preference for textual specificity over analogical generation is a notable feature of its 2023 codification methodology.

89 Detailed reform proposals are deferred to section 10. In broad outline, the proposal is to introduce into the Saudi Code a counterpart to Jordanian art 291, calibrated to the algorithmic and platform-based harms identified in this section.

90 On the Chinese alternative—codified personality rights with strict-liability platform obligations under the 2021 Civil Code—see Peng and Lee (2025) (treating the model as functionally instructive without endorsing it as directly transplantable).

91 US courts have begun to recognise platform amplification as legally distinct from passive hosting in cases involving recommender-system-driven harm; the trend is surveyed in Kadri and West (2025) 540–552; and discussed at the level of practice in industry commentary, eg “Your Algorithm Is Now the Defendant: Why AI Content Moderation Is Creating New Legal Risk for Platforms” (Traverse Legal, October 2025).

92 On the EU’s Article 35 DSA risk-based duties for very large online platforms (VLOPs), and their interaction with civil-liability frameworks, see further section 8 below.

93 On al-sabab al-muntij (the productive cause) and its French ancestor la cause adéquate, see al-Sanhūrī (1952), p. 962–978; for the original German formulation, von Bar (1871).

94 On causation as a normative rather than factual selection, see Hart and Honoré (1985), ch. 1; for the civilian counterpart, al-Sanhūrī (1952), p. 980–995.

95 On distributed and entangled causation in algorithmic environments, see de Cock Buning (2016) and Citron and Solove (2022), p. 820–830. The distinction between sequential and entangled causation is central to the present argument: sequential causation supports doctrinal apportionment in ways that entangled causation does not.

96 On the operational opacity of contemporary recommender algorithms even to their operators, see “Uta Kohl”; the article is authored by Uta Kohl. “Toxic Recommender Algorithms: Immunities, Liabilities and the Regulated Self-Regulation of the Digital Services Act and the Online Safety Act” (2024) Journal of Media Law (advance article); and Klonick (2018), p. 1,635–1,645.

97 On engagement-optimised algorithms producing emergent rather than designed outputs, see DiResta (2024) ch. 4; and the empirical illustration in the TikTok recommender study cited in Domingo and others.

98 On the parameter-distributed and uninterpretable nature of foundation-model “understanding,” see Henderson and others 4–7. The technical inability to predict particular outputs from particular inputs is a feature, not a contingent failure, of large generative architectures.

99 On viral propagation networks and their resistance to single-actor causal analysis, see Chesney and Citron (2019), p. 1773–1779; and DiResta (2024) ch. 4–5.

100 On the calibration of foreseeability to the position of the actor at the moment of the act, see al-Sanhūrī (1952), p. 995–1,010; and for the comparative French treatment, Geneviève Viney, “La responsabilité civile à l’épreuve de l’évolution des sciences et des techniques” RTD civ 2003.681.

101 On the distinction between category-level and instance-level foreseeability in AI tort liability, see Kadri and West (2025), p. 535–540; and the discussion of “model-level” liability in Chesney and Citron (2019), p. 1789–1796.

102 On the evidentiary asymmetry between deepfake plaintiffs and platform/developer defendants, and the resulting pressure for burden-shifting rules, see Citron and Solove (2022), p. 832–840; and Delfino (2024).

103 Saudi civil procedure does not contain a comprehensive discovery regime analogous to that of common-law systems; document-production orders are available but require specific identification of the documents sought, a requirement plaintiffs in algorithmic-causation cases cannot easily meet. The Jordanian position is comparable. See Saudi Code of Civil Procedure (Royal Decree M/1 of 2013), arts 99–104; Jordanian Code of Civil Procedure (Law No 24 of 1988), arts 76–82.

104 On art 35 DSA risk-based duties for very large online platforms, see Regulation (EU) 2022/2065 on a Single Market for Digital Services [2022] OJ L277/1, art 35; for the recent CJEU case-law extending platform controllership beyond the e-Commerce Directive’s safe harbour, see X v Russmedia Digital SRL (2025). The DSA approach. is a regulatory rather than civil-procedure response to multi-causal harm; the two regimes operate in parallel.

105 On counterfactual (“but-for”) causation in classical tort and its limits when applied to multi-actor algorithmic harm, see Hart and Honoré (1985), p. 109–129; and the application to AI systems in Bathaee (2018).

106 On the Chinese model’s integration of personality-rights protection with intermediary obligations under the Civil Code (2021), arts 989–1,039, see Peng and Lee (2025). The Chinese model abolishes the singular-tortfeasor presupposition in favour of a layered-liability scheme, but does so within a civil-law framework whose preconditions differ materially from the Saudi-Jordanian context.

107 Chesney and Citron (2019), p. 1773–1779 propose a “virality index” as a calibration measure for damages quantum in cases of synthetic-media harm; the proposal addresses the third-gap pressure of structural foreseeability through measurement rather than doctrinal redefinition.

108 On the EU AI Act art 50 transparency requirements as a risk-mitigation rather than liability-allocation regime, see European Commission, Code of Practice on Transparency of AI-Generated Content (first draft, 17 December 2025); and the analysis in Bird & Bird, “Taking the EU AI Act to Practice” (March. 2026).

109 A methodological note: doctrinal analysis can identify the gap but cannot itself supply the evidentiary instruments required to close it. The institutional reform implied by the third gap—burden-shifting rules, mandatory algorithmic logging, expert-witness frameworks—lies beyond civil-code amendment proper. The reform proposals in section 10 accordingly distinguish doctrinal from procedural reform.

110 On the institutional preconditions for effective multi-actor liability in the algorithmic context, see Bradford (2023), ch. 6; and the broader regulatory framework in Bradford (2023).

111 Section 10 below proposes (a) a Saudi counterpart to Jordanian art 291, (b) an explicit doctrinal acknowledgment of joint primary liability for layered tortfeasors, and (c) burden-shifting evidentiary rules in deepfake civil proceedings. Items (a) and (b) close the second gap; item (c) addresses the third.

112 I treat the comparative material in this section as functional rather than systematic; the question is which external models offer instructive responses to the gaps identified in sections 5–7, not whether any model is wholesale transplantable. On the methodological commitment, see Zweigert and Kötz (1998), p. 36–46.

113 The selection of the EU, US, and UAE as comparators is not exhaustive. The Chinese model is engaged at section 7.4 above and revisited briefly in section 8.4; the Singaporean and Korean models are noted in passing. The three primary comparators are chosen because they represent the principal regulatory archetypes confronting deepfake harm in 2026, and because the UAE provides a regional bridge between the Saudi-Jordanian framework and the Western regulatory traditions.

114 Regulation (EU) 2024/1689 of the European Parliament and of the Council laying down harmonised rules on artificial intelligence (Artificial Intelligence Act) [2024] OJ L1689/1.

115 AI Act (Regulation (EU) 2024/1689, 2024) art 50(2). The provision applies to providers of “AI systems, including general-purpose AI systems, generating synthetic audio, image, video or text content”; on the first draft Code of Practice operationalising this obligation, see European Commission, First Draft Code of Practice on Transparency of AI-Generated Content (17 December 2025), which proposes a “multilayered marking” approach. Combining metadata embedding, watermarking, and visible labelling.

116 AI Act (Regulation (EU) 2024/1689, 2024) art 50(4): “Deployers of an AI system that generates or manipulates image, audio or video content constituting a deep fake, shall disclose that the content has been artificially generated or manipulated.” The exemption for “evidently artistic, creative, satirical, fictional or analogous” works is set out in the same provision.

117 On the August 2026 entry-into-force date and its implications for compliance design, see Bird & Bird (2026) and Jones Day (2026).

118 Regulation (EU) 2022/2065 art 14 (terms-and-conditions transparency) and art 16 (notice-and-action). On the integration of these provisions with art 35’s systemic-risk regime, see Wilman (2022).

119 DSA (Regulation (EU) 2022/2065, 2022) art 35(1), requiring providers of very large online platforms and very large online search. Engines to “put in place reasonable, proportionate and effective mitigation measures, tailored to the specific systemic risks identified pursuant to Article 34”. Synthetic-media disinformation is expressly identified among the systemic risks under art 34(1)(c).

120 X v Russmedia Digital SRL (2025), paras 41–58. The Grand Chamber’s reasoning departs from earlier cases (e.g., Case C-682/18, YouTube and Cyando, 2021) by emphasising the platform’s active role in the data-processing operation, rather than its passive hosting status.

121 GDPR (Regulation (EU) 2016/679, 2016) art 82(1): “Any person who has suffered material or non-material damage as a result of an infringement of this Regulation shall have the right to receive compensation from the controller or processor for the damage suffered.”

122 Case C-300/21, UI v Österreichische Post AG (2023) ECLI:EU:C:2023:370. The CJEU held that mere infringement of the GDPR does not in itself give rise to compensation under art 82; the claimant must demonstrate actual non-material damage, though no de minimis threshold applies.

123 Lov om kunstneres ophavsret over personlige rettigheder [Act on Artists’ Copyright Over Personal Rights] (Denmark, draft 2025); see Tech Policy Press (2025).

124 On the conceptual departure represented by the Danish proposal, see Bosher (2026).

125 See Wilman (2022) and Bradford (2020).

126 Communications Decency Act of 1996 § 230, codified at 47 USC § 230. On the persistent immunity of US platforms for user-generated content, see Kosseff (2019).

127 On the federal-state regulatory layering in US deepfake regulation, see Kadri and West (2025), p. 528–548; “Mapping AI Policy: Where, Why, and How to Intervene” (Institute for Law & AI, 2026).

128 Ensuring Likeness Voice and Image Security Act of 2024 (ELVIS Act), Tenn Code Ann § 47-25-1101 et seq. Effective 1 July 2024.

129 On the divergent state-level approaches in California, Texas, Tennessee, and New York, see Kadri and West (2025), p. 540–548.

130 Civil Code of the United Arab Emirates, Federal Law No 5 of 1985, art 316: “A person shall be liable for the harm caused by inanimate objects under his custody, unless he proves that the harm occurred for a cause that he could not have prevented.”

131 On the textual proximity of UAE art 316 to Jordanian art 291, see al-Sanhūrī (1952) 1075 ff; and Maḥmūd Aḥmad ‘Alī, al-Masʾūliyya al-madaniyya ‘an ḥirāsat al-ashyāʾ fī dawlat al-Imārāt (Dār al-Nahḍa al-’Arabiyya 2018).

132 UAE Federal Decree-Law No 34 of 2021 on Combating Rumours and Cybercrimes; UAE Federal Decree-Law No 45 of 2021 on the Protection of Personal Data. See Saadeh and Asaad (2022).

133 The UAE National AI Strategy 2031, adopted in 2017 and updated in 2023–2024, operates as a policy framework rather than a binding instrument. See UAE Government (2024).

134 Sumsub (2024) Table 7 (regional breakdown of deepfake fraud incidents); see also Arab News.

135 The DEFIANCE Act’s sectoral coverage is limited to non-consensual intimate imagery; it does not address corporate deepfake harm.

136 The UAE Civil Code’s art 316 provides the closest existing regional analogue to the legislative reform proposed in section 10 for Saudi Arabia.

137 The dual UAE legislation on cybercrime (2021) and personal data protection (2021) operates alongside, not in place of, the Civil Code’s tort framework.

138 Bosher (2026) treats the Danish proposal as a structurally distinct fourth approach. I treat it here as a variant of the personality-rights approach.

139 China’s Civil Code (2021), arts 989–1,039 (personality rights) and arts 1,194–1,197 (network platform liability). See Peng and Lee (2025), p. 11–22.

140 See Peng and Lee (2025).

141 The transferability assessment in this section is preliminary; a fuller calibration is undertaken in section 10.

142 See Reimann (2012), pp. 13–34.

143 Specifically, transparency obligations on providers of generative AI systems (modelled on AI Act art 50(2)) and labelling duties on deployers (modelled on art 50(4)) can be enacted as administrative regulations under existing rule-making authority.

144 The systemic-risk obligations of DSA art 35 presuppose the existence of a regulatory authority with both technical capacity and legal authority. The Saudi CST and Jordanian Telecommunications Regulatory Commission would need expansion of mandate to perform this function.

145 The Russmedia approach. Presupposes a private right of action analogous to GDPR art 82. Both the Saudi PDPL (2021) and the Jordanian PDPL (2023) currently lack such. a right.

146 On legal-transplant theory, see Watson (1993) and Legrand (1997).

147 The empirical evidence cited at sections 5.3 and 7.2 above provides functional grounds for the same calibration: the dominant deepfake litigation profile in 2026 is structurally addressed by the EU model in ways that the US model does not match.

148 The legislative proposals in section 10 select the EU framework as principal template, while drawing supplementary doctrinal elements from the UAE’s art 316 and from Chinese-style burden-shifting rules.

149 On maqāṣid al-sharī’a generally, see Abū Ḥāmid al-Ghazālī, al-Mustaṣfā min ‘ilm al-uṣūl (Muḥammad ‘Abd al-Salām ‘Abd al-Shāfī ed., Dār al-Kutub al-’Ilmiyya 1,413 AH) vol 1, 286–287; Abū Isḥāq al-Shāṭibī, al-Muwāfaqāt (Mashhūr Ḥasan Āl Salmān ed., Dār Ibn ‘Affān 1997) vol 2, 17–25; Muḥammad al-Ṭāhir Ibn ‘Āshūr, Maqāṣid al-sharī’a al-Islāmiyya (Muḥammad al-Ṭāhir al-Mīsāwī ed., al-Basāʾir 2001).

150 On ḥifẓ al-’irḍ as an objective, see al-Shāṭibī (1997) vol 2, 19–21; and “Freeing Maqāṣid and Maṣlaḥa” (2016), pp. 7–8.

151 Qurʾān 24:4 (the prescribed punishment for false accusation of unchastity, qadhf); 49:11–12 (the prohibitions of mocking, defamatory naming, suspicion, spying, and backbiting).

152 See al-Qurṭubī, al-Jāmi’ li-aḥkām al-Qurʾān (Muʾassasat al-Risāla 2006) vol 15, 154–181; ‘Abd al-Qādir ‘Awda, al-Tashrī’ al-jināʾī al-Islāmī muqāranan bi-l-qānūn al-waḍ’ī (Muʾassasat al-Risāla 1992) vol 2, 459–485.

153 See Ibn Manẓūr, Lisān al-’Arab (Dār Ṣādir 1,414 AH) vol 13, 53; al-Nawawī, Riyāḍ al-ṣāliḥīn (Shu’ayb al-Arnāʾūṭ ed., Muʾassasat al-Risāla 1998), bāb taḥrīm al-ghība wa-l-namīma.

154 European personality rights—including the German Persönlichkeitsrecht and the French droit à l’image—operate as private-law entitlements grounded in autonomy and dignity. Ḥifẓ al-’irḍ operates as a public-order objective whose private-law expression is mediated through specific provisions (ḍamān al-ḍarar al-adabī).

155 See al-Shāṭibī (1997) vol 2, 25–30; Hallaq (1997), ch. 5.

156 On al-fatāwā al-jamā’iyya, see Wahbah al-Zuḥaylī, Uṣūl al-fiqh al-Islāmī (2nd edn, Dār al-Fikr 1986) vol 2, 1,163–1,180.

157 See al-Qaraḍāwī (1990), pp. 100–118.

158 See “Islamic Legal Analysis on the Protection of Vulnerable Age Groups in Online Gender-Based Violence” (2024) Journal of Ecohumanism; “Preventing AI Deepfake Abuse: An Islamic Ethics Framework” (2025), advance article.

159 See al-Suyūṭī, al-Ashbāh wa-l-naẓāʾir (Dār al-Kutub al-’Ilmiyya 1990) 87.

160 See Opwis (2010), pp. 285–330; Hallaq (2009) 162–180.

161 The reform proposals are calibrated to operate within the existing constitutional architecture of both Saudi Arabia and Jordan.

162 See al-Qaraḍāwī (1991), pp. 107–122; al-Raysūnī (1995), pp. 248–265.

163 See al-Mu’ajjal (1442AH) 156–178.

164 See al-Zu’bī (2018) 24–36; Sulṭān (2018) 18–24.

165 Specifically: maqāṣid-level reasoning supports (a) reading “natural person” in art 138(2) of the Saudi code in light of ḥifẓ al-’irḍ-grounded protection of corporate-style endowments; (b) interpreting the wider phrasing of Jordanian art 267 in line with its textual breadth; and (c) treating the platform-and-developer custody of generative systems as triggering analogous ḍamān-style obligations.

166 See Kamali (2002) and Vogel (2000), ch. 9.

167 The Sharī’a foundation does not dictate a particular external model; it constrains the field of legitimate options to those that protect ḥifẓ al-’irḍ and prevent buhtān-type harm.

168 Section 10 below converts the comparative observations of section 8 and the normative observations of this section into specific legislative-reform proposals.

169 Article 138(2), Civil Transactions Law (Saudi Arabia, 2023). Current text: “Moral harm includes the physical or psychological suffering inflicted on the natural person (al-shakhṣ dhū al-ṣifa al-ṭabī’iyya) as a result of injury to his body, freedom, honour, reputation, or social standing.”

170 Proposed Arabic formulation: “al-ḍarar al-adabī yashmalu al-alama al-jasadī aw al-nafsī alladhī yulḥaq al-shakhṣ ṭabī’iyyan kāna aw i’tibāriyyan, natījata al-i’tidāʾ ‘alā jasadihi aw ḥurriyyatihi aw ‘irḍihi aw sum’atihi aw markazihi al-ijtimā’ī, wa-yashmalu fī ḥālat al-shakhṣ al-i’tibārī al-i’tidāʾ ‘alā sum’atihi al-tijāriyya aw raʾsmālihi al-i’tibārī”.

171 The phrase “reputational capital” (raʾsmāl i’tibārī) is a deliberate doctrinal coinage.

172 Comparative drafting precedent: French. Civil Code art 1240; Egyptian Civil Code (Egypt) (1948) art 222; UAE Civil Code (United Arab Emirates) (1985) art 290.

173 See al-Zu’bī (2018), p. 256–274.

174 A new Article 124 bis to the Civil Transactions Law, modelled on Jordanian art 291 and UAE art 316.

175 Proposed Arabic formulation: “Yusʾalu kullu man kāna taḥta taṣarrufihi ashyāʾ aw anẓima tatatallabu ‘ināyatan khāṣṣatan li-l-wiqāya min ḍararihā, bi-mā fī dhālika al-anẓima al-tiqniyya wa-l-khwārizmiyya al-tūlīdiyya, ‘an al-ḍarar alladhī tuḥdithuhu, mā lam yuthbit anna wuqū’a al-ḍarar kāna bi-sabab lā yad lahu fīhi”.

176 On the calibration considerations, see Bacache (2020) and Loiseau (2019).

177 Insertion of a private right of action analogous to GDPR art 82, modelled on the X v Russmedia Digital SRL (2025) framework.

178 The Jordanian PDPL (Law No 24 of 2023) requires parallel amendment.

179 Structurally analogous to res ipsa loquitur in common-law systems and to the French présomption de responsabilité. See Viney et al. (2013) nos 685 ff.

180 Proposed Arabic formulation: “Idhā tawaffara al-mud’ī ‘alā dalīlin awwalī yujāwizu ‘atabat al-iḥtimāl al-rājiḥ ‘alā wujūdi ḍararin nāshiʾ ‘an istikhdāmi nāẓim takhliqī ittiṣālan sababiyyan li-mud’an ‘alayhi, intaqala ‘ibʾ al-ithbāt fī mā yata’allaqu bi-ghiyābi al-sababiyya aw bi-ittikhādhi al-tadābīr al-fanniyya al-mulāʾima ilā al-mud’an ‘alayh”.

181 See Saudi Code of Civil Procedure arts 99–104; Jordanian Code of Civil Procedure arts 76–82.

182 Modelled on AI Act art 50(2)–(4) and on the December 2025 draft Code of Practice.

183 The proposed administrative regulation should require: (a) machine-readable marking of AI-generated content; (b) visible labelling of deepfakes for public-interest content; (c) systemic-risk assessment by VLOP-equivalent platforms; and (d) cooperation with the regulatory authority on takedown and remediation.

184 Items (a) and (b) (the civil-code amendments) require legislative process. Items (c) and (d) (data-protection amendment, administrative regulation) can proceed in parallel under existing rule-making authority. Item (e) (the burden-shifting rule) requires either legislative amendment or formal procedural directive.

185 The package is internally complementary.

186 The Jordanian reform sequence is functionally parallel: judicial reorientation of art 267, targeted exegesis of art 291, data-protection law amendment, and a procedural directive on burden-shifting.

187 Implementation challenges include: regulator capacity, judicial training in algorithmic-causation evidence, cross-border enforcement, and harmonisation of the two national reform tracks.

188 The reform proposals are calibrated to the gaps identified in sections 5–7, the comparative material developed in section 8, and the normative foundation articulated in section 9.

## Appendix

**Table A1:** Comparative calibration matrix.

Criterion	EU	US	UAE	China
Regulatory model	Layered regulation	Sectoral/statutory patchwork	Regional civil-law bridge	Personality-rights model
Deepfake transparency	Explicit AI/disclosure duties	Mainly specific statutes	General cyber/media controls	Platform and content controls
Platform responsibility	Risk-based duties for major platforms	Limited by intermediary immunity debates	Developing regulatory duties	Stronger intermediary obligations
Private civil remedy	Data-protection and tort routes	Varies by state and subject matter	General civil-code remedies	Personality-rights claims
Fit with Saudi/Jordanian law	High as a structured reference	Medium/low as a transplant	Medium/high regional relevance	Selective usefulness only
Main limitation	Complex institutional design	Fragmented and less civilian	Less deepfake-specific	Different constitutional/regulatory setting

This matrix summarises the functional comparison used in the article. It is not a ranking of legal systems. Its purpose is to show which external models are most compatible with the doctrinal gaps identified in the Saudi-Jordanian framework.
